# Plasma proteomic biomarkers as mediators for the associations between frailty phenotypes and chronic respiratory diseases

**DOI:** 10.1007/s11357-026-02099-w

**Published:** 2026-02-04

**Authors:** Yujia Bao, Shenghao Yuan, Jiawei Gu, Chen Shen, Linli Chen, Yibin Zhou, Jinjun Ran

**Affiliations:** 1 Minhang District Center for Disease Control and Prevention, Shanghai, 201101 China; 2 School of Public Health, Shanghai Jiao Tong University School of Medicine, Shanghai, 200025 China; 3 Department of Epidemiology and Biostatistics, School of Public Health, MRC Centre for Environment and Health, Imperial College London, London, UK

**Keywords:** Chronic respiratory disease, Cohort study, Frailty, Proteomic signatures

## Abstract

Frailty, a hallmark of systemic vulnerability in aging populations, is increasingly recognized in the clinical management of chronic respiratory diseases (CRDs). Molecular mechanism underpinning the relationships remain insufficiently elucidated. This study hence aimed to investigate whether proteomic biomarkers—circulating plasma proteins reflecting systemic inflammation, metabolism, and tissue remodeling—are associated with CRDs and may serve as potential mediators of the observed links. We analyzed data from a population-based cohort of 22,802 adults with proteomic measurements. Frailty was assessed by three phenotypes, including frailty index, physical frailty, and psychological frailty. Related-proteomic signatures were estimated by both linear and elastic regression models. Cox regression models were applied to explore the associations of frailty phenotypes and their proteomic signatures with incident CRDs, including asthma, chronic obstructive pulmonary disease (COPD), and idiopathic pulmonary fibrosis (IPF), as well as lung function outcomes, with full adjustments for potential confounders. Furthermore, mediation analyses were conducted to explore underlying mechanisms, complemented by pathway enrichment analyses to reveal relevant biological functions. Over a median follow-up of 13.2 years, 617 participants developed asthma, 701 developed COPD, and 228 developed IPF. Higher frailty index was associated with elevated risks of asthma (HR 1.95, 95% CI 1.64 to 2.31) and COPD (2.02, 1.71 to 2.38). Corresponding proteomic signature also related to increased risks of asthma (HR 1.22, 1.11 to 1.34) and COPD (1.65, 1.53 to 1.78). Mediation analysis suggested that the frailty index partially mediated the association with COPD, accounting for 26.0% (95% CI: 18.7 to 37.5%) of the total effect, respectively, particularly for GDF15, WFDC2, and PLAUR. Pathway enrichment analysis showed that these mediating proteins were predominantly involved in immune activation, inflammatory signaling, and metabolic stress responses. Frailty phenotypes contribute to elevated CRDs risks, partly through proteomic dysregulation in inflammatory and metabolic pathways.

**Graphic Abstract:**

**Figure:**
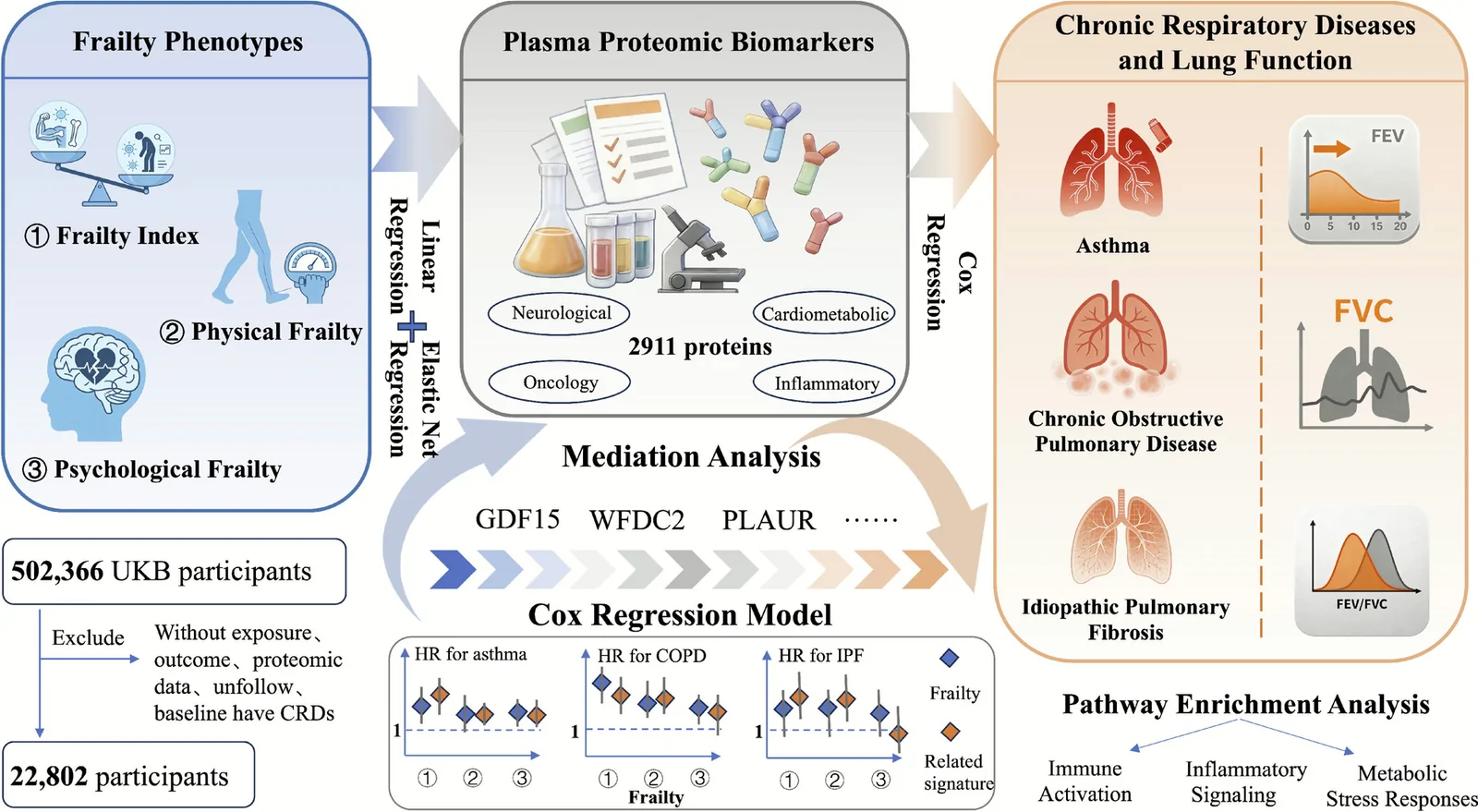

**Supplementary Information:**

The online version contains supplementary material available at https://doi.org/10.1007/s11357-026-02099-w.

## Introduction

Inexorable population aging and the emergence of precision medicine have not only reshaped global health systems but also catalyzed a paradigm shift in public health strategies toward personalized risk stratification and long-term care planning, especially in vulnerable populations [1]. Frailty is globally recognized as a multidimensional clinical syndrome marked by a progressive decline in physiological reserves and a reduced capacity to withstand internal or external stressors [2]. The shift of public health paradigm has elevated the importance of frailty assessment across disciplines, positioning it as a core consideration in the management of high-risk populations burdened by multimorbidity [3]. The European Innovation Partnership on Active and Healthy Ageing acknowledged frailty as a critical consideration in the clinical management and care planning of individuals with chronic respiratory diseases (CRDs) [4]. In 2023, the European Respiratory Society (ERS) issued a statement emphasizing the importance of identifying and assessing frailty in the management of chronic lung diseases [5]. As a preventable and reversible condition, frailty demands deeper insight into its link with CRDs to advance precision care and integrated prevention in high-risk groups [6, 7].

Despite increasing evidence supporting the bidirectional relationships between frailty and CRDs, most studies focus only on physical aspects, overlooking multidimensional constructs like psychological frailty [8–10]. Furthermore, the biological mechanisms connecting frailty and CRDs remain insufficiently characterized. Emerging evidence suggested that proteomic signatures may capture early biological perturbations and offer insights into the underlying pathophysiology of frailty and respiratory conditions [11, 12]. These signatures are generated from high-throughput proteomic profiling platforms, which provide comprehensive coverage of circulating proteins. Large-scale proteomic profiling based on the Olink proximity extension assay (PEA) platform enables simultaneous quantification of thousands of plasma proteins in relative units (NPX), capturing molecular signatures of systemic inflammation, metabolic dysfunction, immune dysregulation, and tissue remodeling—hallmarks implicated in both frailty and CRDs [13, 14]. However, population-based evidence on the potential mediating role of proteins in the relationship between frailty and chronic respiratory diseases remains scarce, particularly in aging societies facing increasing multimorbidity and healthcare demands.

To fill the gap, the study assessed the longitudinal associations of frailty phenotypes and proteomic alterations with the incidence risks of CRDs, and further investigated the mediating role of proteins in the frailty–lung disease axis. Moreover, we identified shared molecular pathways between frailty and respiratory dysfunction using co-expression networks and pathway enrichment.

## Methods

### Study design and participants

**Fig. 1:**
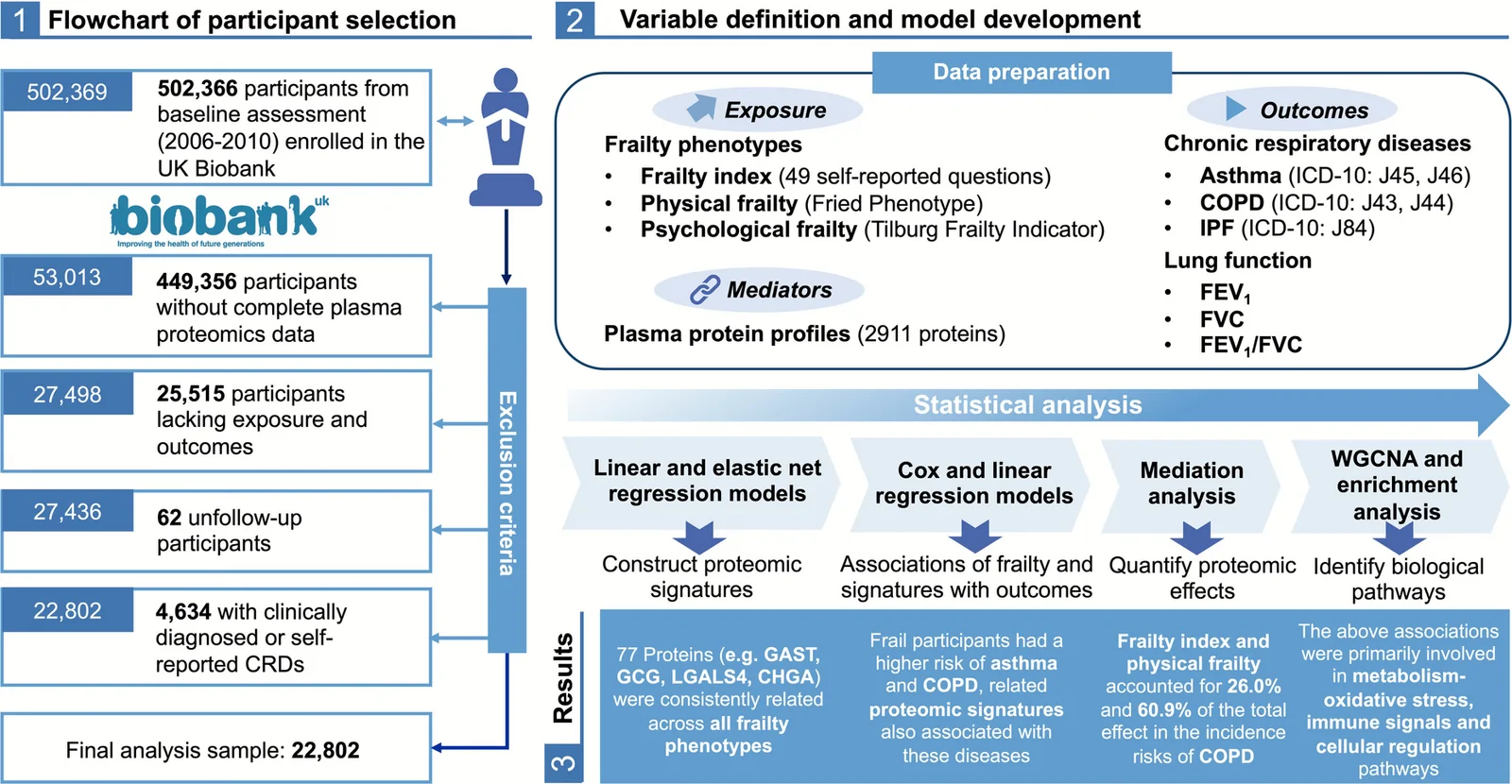
Study workflow. Left: participant flowchart in the analysis. Top right: Multiple exposures (three frailty phenotypes: frailty index, physical frailty, and psychological frailty), outcomes (chronic respiratory diseases and lung function), and mediators (2911 proteins) in this study. Bottom right: the main analyses and results

This study utilized data from the UK Biobank, a large-scale, population-based prospective cohort involving over 500,000 individuals aged 37 to 73 at the time of enrollment, which took place between 2006 and 2010. Baseline evaluations included self-administered touchscreen surveys, verbal interviews, electronic records, physical health measurements, and biological specimens [15]. Of the 53,013 individuals with complete plasma proteomics data, we excluded 25,515 participants lacking exposure and outcomes, 62 participants who were unfollow-up, and 4,634 with clinically diagnosed or self-reported respiratory diseases, the final analysis included 22,802 participants (Fig. 1). Written informed consent was obtained from all participants, with ethical approval granted by the North West Multicenter Research Ethics Committee (Reference: 11/NW/03820). The study was conducted under the application number 99001 (2023–01–31 to 2026–01–31).

### Exposure measurement

Frailty was evaluated at baseline using multiple frailty domains, namely frailty index, physical frailty, and psychological frailty, which were widely used in previous clinical studies [16, 17]. The questionnaires used to diagnose each frailty domain are described in the eMethods and Table S1-S3. Frailty index and physical frailty were categorized into non-frail (< 0.12/0 scores), pre-frail (0.12–0.24/1–2 scores), and frail (> 0.24/≥ 3 scores) groups based on established cut-off values [17–19]. Due to the limited sample size in the frail group under the three-level classification of frailty index and physical frailty, prefrail and frail were combined versus non-frail (as the reference) when estimating the binary classification effects. Since there is no official recommended classification method for psychological frailty, we used quartiles as the three-category cut-off values and the median for dichotomy.

### Outcome ascertainment

The primary outcomes of the study were incident CRDs, including asthma, chronic obstructive pulmonary disease (COPD), and idiopathic pulmonary fibrosis (IPF). Disease diagnoses were obtained from hospital episode databases, which were linked to electronic health records and classified using the International Classification of Diseases (ICD-9 and ICD-10) codes. The ICD-10 codes of each disease were as follows: asthma (J45, J46), COPD (J43, J44), and IPF (J84) (Table S4). The endpoint event was defined as the earliest occurrence of any CRDs, death, or end of follow-up (November 30, 2022), whichever came first. The duration of follow-up was computed from the date of baseline recruitment to the endpoint event. Furthermore, FEV_1_, FVC, and FEV_1_/FVC were included as the secondary outcomes to assess the lung function, which were normalized using z-score transformation before analysis (eMethods).

### Proteomic profiling

The plasma proteomic profiles of approximately 54,000 participants were characterized by the UK Biobank Pharma Proteomics Project. Plasma samples were collected at baseline and stored at –80 °C and transported on dry ice to the Olink Analysis Service for processing. Detailed information is shown in eMethods. Among 2,923 distinct proteins, 20% or more with missing data were excluded, resulting in a final set of 2,911 proteins. For those missing less than 20%, we imputed them using the mean value. Additional details are provided in Table S5.

### Covariates

Socio-demographic factors (age, sex, ethnicity, index of multiple deprivation [IMD], income, and education), body mass index (BMI), waist-to-hip ratio (WHR), occupational exposure, lifestyle factors (pack years of smoking, never smoking, moderate drinking, regular physical activity, healthy sleep pattern, and healthy diet), C-reactive protein, environmental factors (noise pollution, proximity to roadway, baseline exposure of PM_2.5_ and NO_2_), and prevalent diseases (hypertension and diabetes), which could potentially confound the relationships between frailty and CRDs, were included as covariates [20–22]. Proteomic variability was minimized by adjusting for fasting duration (hours), sampling season (summer/autumn or winter/spring), and sample age (days). To address missing data in covariates, multiple imputation was performed using 20 iterations. The covariates are described in the eMethods and Table S6-S8.

### Statistical analyses

Descriptive statistics for baseline characteristics included mean ± standard deviation (SD) for continuous variables with approximately normal distributions and median (Q1, Q3) for skewed variables. Categorical variables were summarized as frequencies and percentages. To derive proteomic signatures corresponding to three frailty domains, we implemented a two-stage analytical approach. In the first stage, multiple linear regression models were used to examine associations between each protein and frailty, applying false discovery rate (FDR) correction and adjusting for socio-demographic factors, BMI, WHR, lifestyle factors, fasting time, season, and sample age. In the second stage, the data were randomly partitioned, with 70% allocated for model training and 30% reserved for validation. Proteins that met the significance threshold (*P* < 0.05) were subjected to the elastic net regression in the training dataset, a hybrid method combining least absolute shrinkage and selection operator regression and Ridge regression (eMethods). The proteomic signature was computed as a weighted sum of the selected proteins and validated in the testing dataset [23]. The model in both datasets suggested high discriminatory ability and reliable generalization performance (Table S9). Moreover, for proteins significantly associated with each frailty domain, we explore the relevant pathways and biological functions linked to the corresponding target genes using the Kyoto Encyclopedia of Genes and Genomes and Gene Ontology databases.

We employed Cox proportional hazards regression models to investigate hazard ratios (HRs) and 95% confidence intervals (CIs) for the associations of three frailty domains and related-proteomic signatures with the incident CRDs, using follow-up time as the underlying time scale. The proportional hazards assumption was evaluated using Schoenfeld residuals, and no significant violations were observed. Furthermore, we utilized multiple linear regression models to estimate the associations between frailty and lung function, with the results shown as βs and 95% CI. Cox and linear regression models with varying covariate adjustments were constructed (eMethods). In addition, associations of individual proteins with CRDs and lung function were both explored using the multivariable models. We further performed mediation analyses and calculated the estimated mediating proportions to evaluate the potential role of proteomic signatures and individual proteins in the associations. Additionally, to gain insights into the biological functions of frailty-related proteins, we applied weighted correlation network analysis (WGCNA) to identify clusters of co-expressed proteins. Subsequent pathway enrichment analyses were conducted on these protein modules to explore their functional relevance. We did several sensitivity analyses to validate the robustness of the results. First, we restricted the analysis to Whites due to the high proportion (~ 97%) of Whites in the cohort. Second, preclinical or undiagnosed respiratory dysfunction at baseline could theoretically manifest as reduced physical capacity or systemic dysregulation, thereby inflating frailty and strengthening the observed associations. To minimize reverse causation, individuals who developed CRDs within the first two years of follow-up were excluded. Third, to mitigate potential confounding effects of the COVID-19 pandemic, follow-up was censored on December 31, 2019. Fourth, we tested the influence of the definitions of confounding factors by replacing moderate drinking with never drinking, and replacing education, income, and IMD with socioeconomic status. Finally, we employed the Fine-Gray model to account for competing risks.

All statistical analyses were conducted using R version 4.4.2, with statistical significance defined as a two-sided *P*-value less than 0.05. Elastic net regression was conducted using the R package “glmnet”; pathway enrichment analyses with “clusterProfiler”; mediation analyses with “mediation”; multiple imputations using “mice”; and weighted correlation network analysis through “WGCNA”. Protein interaction networks were visualized using Cytoscape.

## Results

### Study participants

A total of 22,802 participants with available proteomic data were included in the study (mean age: 57.6 ± 8.0 years; 51.8% women). Baseline characteristics across the incident diseases and frailty index are summarized in Table 1 and Table S10-S12. During 13.2 years of follow-up, incident cases were identified in 617 participants for asthma, 701 for COPD, and 228 for IPF. Of the 22,802 participants, 1.6% (N = 365) met the frail criteria for frailty index, 5.4% (N = 1223) were classified as frail according to the physical frailty, and 42.3% (N = 9636) were defined as frail for psychological frailty.

**Table 1:** Baseline characteristics of included participants grouped by incident diseases

Characteristic	All participants (*N* = 22,802)	Incident diseases (Yes)		
		Asthma (*N* = 617)	COPD (*N* = 701)	IPF (*N* = 228)
Age, year	57.6 (8.0)	58.6 (7.7)	61.6 (6.2)	62.5 (6.2)
Female	11,813 (51.8%)	352 (57.1%)	289 (41.2%)	84 (36.8%)
IMD^a^	12.4 (6.9, 22.6)	13.5 (7.4, 24.7)	18.0 (9.5, 33.2)	14.9 (7.8, 70.7)
Low income^b^	11,791 (51.7%)	359 (58.2%)	513 (73.2%)	146 (64.0%)
Low education^c^	8671 (38.0%)	260 (42.1%)	409 (58.3%)	107 (46.9%)
White ethnic	21,708 (95.2%)	584 (94.7%)	684 (97.6%)	219 (96.1%)
BMI	27.6 (4.7)	28.9 (5.2)	28.2 (5.0)	28.4 (5.0)
Poor WHR^d^	11,901 (52.2%)	352 (57.1%)	492 (70.2%)	157 (68.9%)
Occupational exposure^e^	2353 (10.3%)	74 (12.0%)	75 (10.7%)	33 (14.5%)
Pack years of smoking	20.6 (11.1, 33.5)	21.9 (13.0, 33.8)	30.3 (18.0,45.5)	23.6 (13.5,37.5)
Healthy lifestyle^f^				
Never smoking	12,247 (53.7%)	314 (50.9%)	119 (17.0%)	73 (32.0%)
Moderate drinking	15,255 (66.9%)	420 (68.1%)	426 (60.8%)	142 (62.3%)
Healthy sleep pattern	11,883 (52.1%)	287 (46.5%)	318 (45.4%)	114 (50.0%)
Regular physical activity	12,095 (53.0%)	316 (51.2%)	362 (51.6%)	115 (50.4%)
Healthy diet	8964 (39.3%)	245 (39.7%)	204 (29.1%)	88 (38.6%)
Prevalence of comorbidities				
Hypertension	8935 (39.2%)	274 (44.4%)	369 (52.6%)	115 (50.4%)
Diabetes	1579 (6.9%)	43 (7.0%)	76 (10.8%)	26 (11.4%)
C-reactive protein	2.53 (4.2)	3.29 (5.4)	4.02 (6.0)	4.14 (7.8)
Environmental factors^g^				
Noise pollution (*L*_*den*_), dB	56.1 (4.3)	56.1 (4.3)	56.1 (4.2)	56.3 (4.5)
Proximity to roadways	1642 (7.2%)	43 (7.0%)	50 (7.1%)	20 (8.8%)
PM_2.5_, μg/m^3^	10.7 (2.0)	10.8 (1.8)	10.8 (1.9)	10.5 (1.9)
NO_2_, μg/m^3^	20.4 (6.4)	20.6 (6.2)	21.5 (6.5)	20.2 (6.3)

Abbreviations *COPD* chronic obstructive pulmonary disease, *IPF* idiopathic pulmonary fibrosis, *IMD* index of multiple deprivation, *BMI* body mass index, *WHR* waist-hip ratio

^a^IMD scores offer a detailed view of deprivation. Domains for IMD calculation: crime score, community safety score, education score, employment score, health score, housing score, income score, living environment score, access to services score, physical environment score

^b^Participants with college or university degree, other professional qualifications, and NVQ or HND or HNC or equivalent were defined as high education, and those with A levels/AS levels or equivalent, O levels/GCSE or equivalent, CSE or equivalent, and none of the above were defined as low education

^c^The income was categorized into low and high levels based on £31,000

^d^Waist-to-hip ratio was calculated through waist/hip measurement and was categorized into poor (≥ 0.9 for men and ≥ 0.85 for women) and ideal

^e^High-risk occupations for respiratory diseases are shown in Supplementary Table 7

^f^The details of healthy lifestyle could be found in Supplementary Methods

^g^The *L*_*den*_ (weighted average noise sound level for 24 h, with a penalty of 5 for evening hours and 10 dB for night hours) was a major indicator of noise pollution. The inverse distance was derived based upon a local road network with traffic intensity greater than 5000 motor vehicles per 24 h. Annual average concentration of PM_2.5_ and NO_2_ were calculated from 2006 to 2010

### Identifying proteins associated with exposure and outcome

**Fig. 2:**
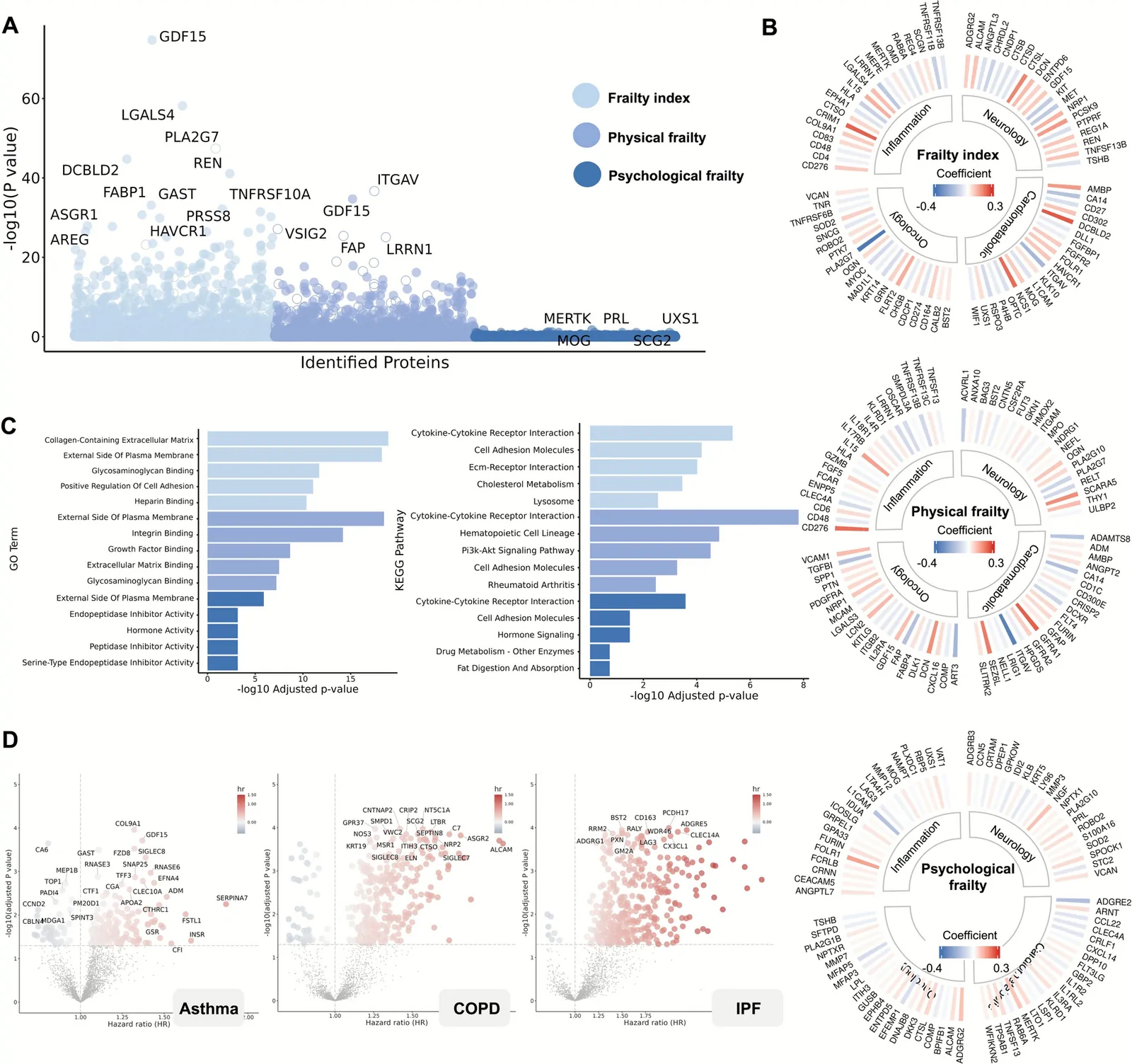
Proteome-wide associations with exposures and outcomes. **A** A Manhattan-style plot illustrating the associations between individual protein abundances and each frailty phenotype, derived from linear regression models adjusted for socio-demographic variables, BMI, WHR, lifestyle factors, fasting duration, season, and sample collection age. **B** Bar plots of β coefficients from the regression analyses between selected proteomic biomarkers and each frailty phenotype. Biomarkers were identified using elastic net regression and used to construct proteomic signature scores for each phenotype. Results are categorized into four proteomic panels: inflammation, cardiometabolic, oncology, and neurology. **C** The top five enriched GO terms and KEGG pathways corresponding to proteins associated with each frailty phenotype. **D** Volcano plots showing associations between individual proteins and the risk of CRDs, estimated using Cox proportional hazards models. Results are expressed as HR, where HR > 1 indicates a detrimental association

Linear regression identified 1197, 807, and 240 proteins that showed significant associations with frailty index, physical frailty, and psychological frailty, respectively (Fig. 2A and Table S13-S16). After FDR correction, 141 to 289 proteins were selected to calculate the protein signatures for three frailty phenotypes through elastic net regression (Fig. 2B and Table S17-S18). Of note, there were 77 proteins that were consistently related across all frailty phenotypes. Pathway enrichment showed that the identified proteins were mainly involved in immunity, inflammation, signal transduction, cell adhesion, and metabolism (Fig. 2C, Fig. S1, and Table S19-S20). Furthermore, we found 222, 710, and 758 proteins remained associated with the incidence risks of asthma, COPD, and IPF (Fig. 2D and Table S21). There were also 589 to 1343 proteins that showed significant relationships with lung function (Fig. S2 and Table S22).

### Frailty, proteomics, and their associations with primary and secondary outcomes

**Fig. 3:**
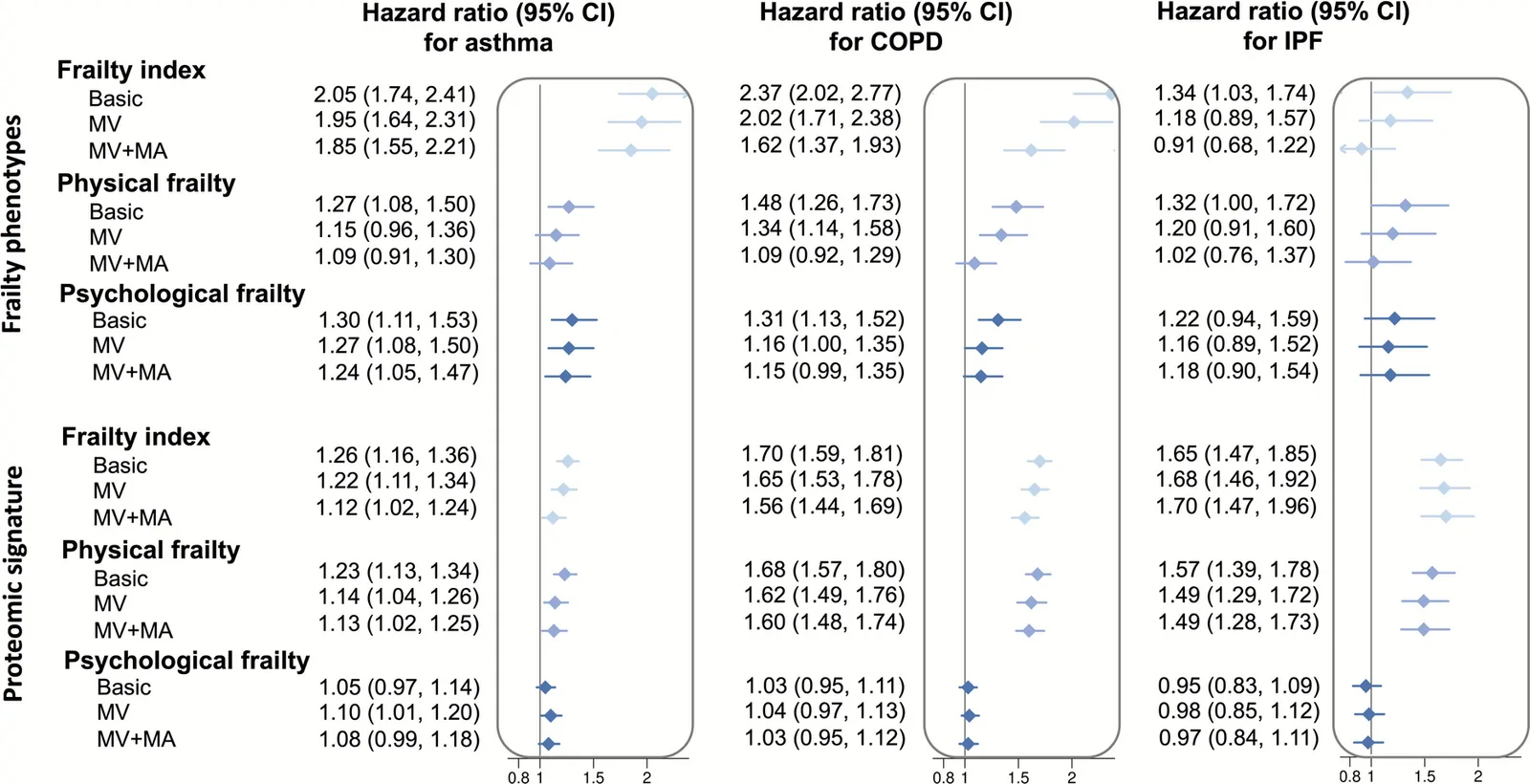
Associations of frailty phenotypes and proteomic signatures with incident CRDs risk. Forest plots showing the associations between frailty phenotypes and their related proteomic signatures with incident risk of CRDs among all participants estimated by Cox regression models. The basic model adjusted for socio-demographic factors. The multivariable model (MV) additionally adjusted for BMI, WHR, occupational exposure, and lifestyle factors, C-reactive proteins, environmental factors, and prevalent diseases. The mutually adjusted model (MV + MA) included both frailty phenotypes and its corresponding proteomic signature. Results are presented as HRs and corresponding 95% CIs

In the multivariable models, compared with non-frail, frail participants (frail and pre-frail combined) had higher risks of asthma (frailty index: HR 1.95, 95% CI 1.64 to 2.31; psychological frailty: 1.27, 1.08 to 1.50) and COPD (frailty index: 2.02, 1.71 to 2.38; psychological frailty: 1.16, 1.00 to 1.35), while physical frailty was only associated with COPD (1.34, 1.14 to 1.58) (Fig. 3 and Table S23-S25). Similarly, related-proteomic signatures demonstrated significant associations with corresponding diseases, yielding HRs of 1.22 (1.11 to 1.34) for frailty index and asthma, 1.65 (1.53 to 1.78) for frailty index and COPD, which remained statistically significant in the mutually adjusted models. For lung function, frailty index and related-proteomic signatures were both negatively associated with FEV_1_ (frailty index: β − 0.09, 95% CI − 0.11 to − 0.07; proteomic signatures: − 0.09, − 0.10 to − 0.07), FVC (frailty index: − 0.08, − 0.10 to − 0.06; proteomic signatures: − 0.07, − 0.08 to − 0.06), and FEV_1_/FVC (frailty index: − 0.03, − 0.06 to − 0.01; proteomic signatures: − 0.05, − 0.07 to − 0.04) in the multivariable models (Fig. S3 and Table S26-S28). No significant sex-specific differences were observed for any frailty–respiratory disease associations (all P for interaction > 0.05). However, age-stratified analyses revealed significant heterogeneity in proteomic signatures. Specifically, the frailty index–proteomic signature showed a stronger association with incident COPD among participants aged < 60 years (1.85, 95% CI: 1.70 to 2.01) than among those aged ≥ 60 years (1.56, 95% CI: 1.40 to 1.74; P for interaction = 0.048) (Table S29-34). The sensitivity analyses showed the robustness of our results (Table S35).

### Mediation analyses and interaction networks of proteins

**Fig. 4:**
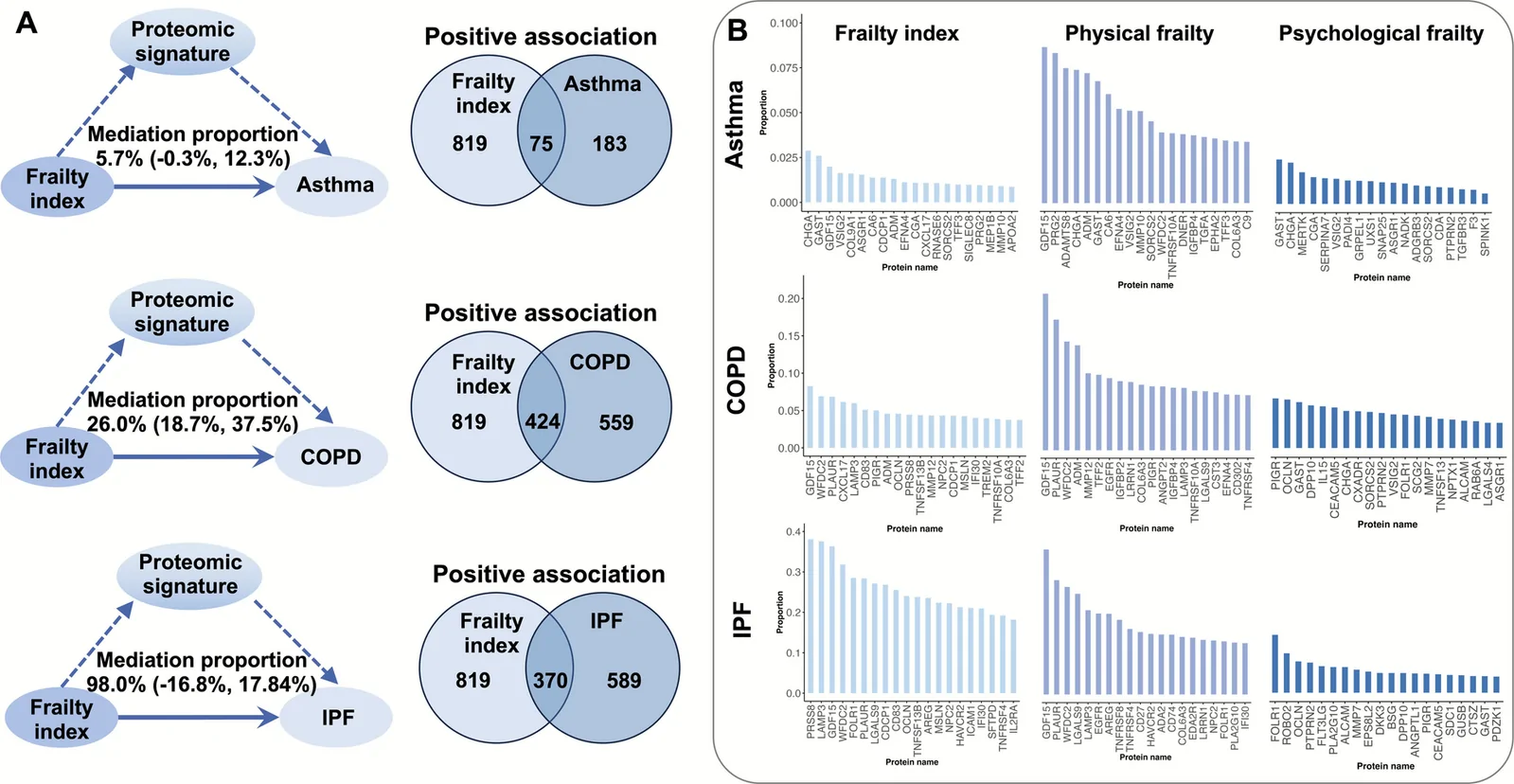
Mediation analysis of proteomic signatures and individual proteins on the relationship between frailty phenotypes and CRDs. **A** the mediation effects of the frailty index-related proteomic signatures on the associations of frailty index with incident CRDs risk, results are presented as mediation proportion derived from mediation analysis. Venn diagram showing the overlap of proteins positively associated with frailty index and three CRDs. The overlapping area indicates proteins jointly associated with both frailty index and the disease. **B** bar plot showing the top 10 proteins ranked by the magnitude of their mediation effect for the individual proteins on the associations of frailty phenotypes with incident CRDs risk. All models were adjusted for socio-demographic factors, BMI, WHR, occupational exposure, and lifestyle factors, C-reactive proteins, environmental factors, and prevalent diseases

**Fig. 5:**
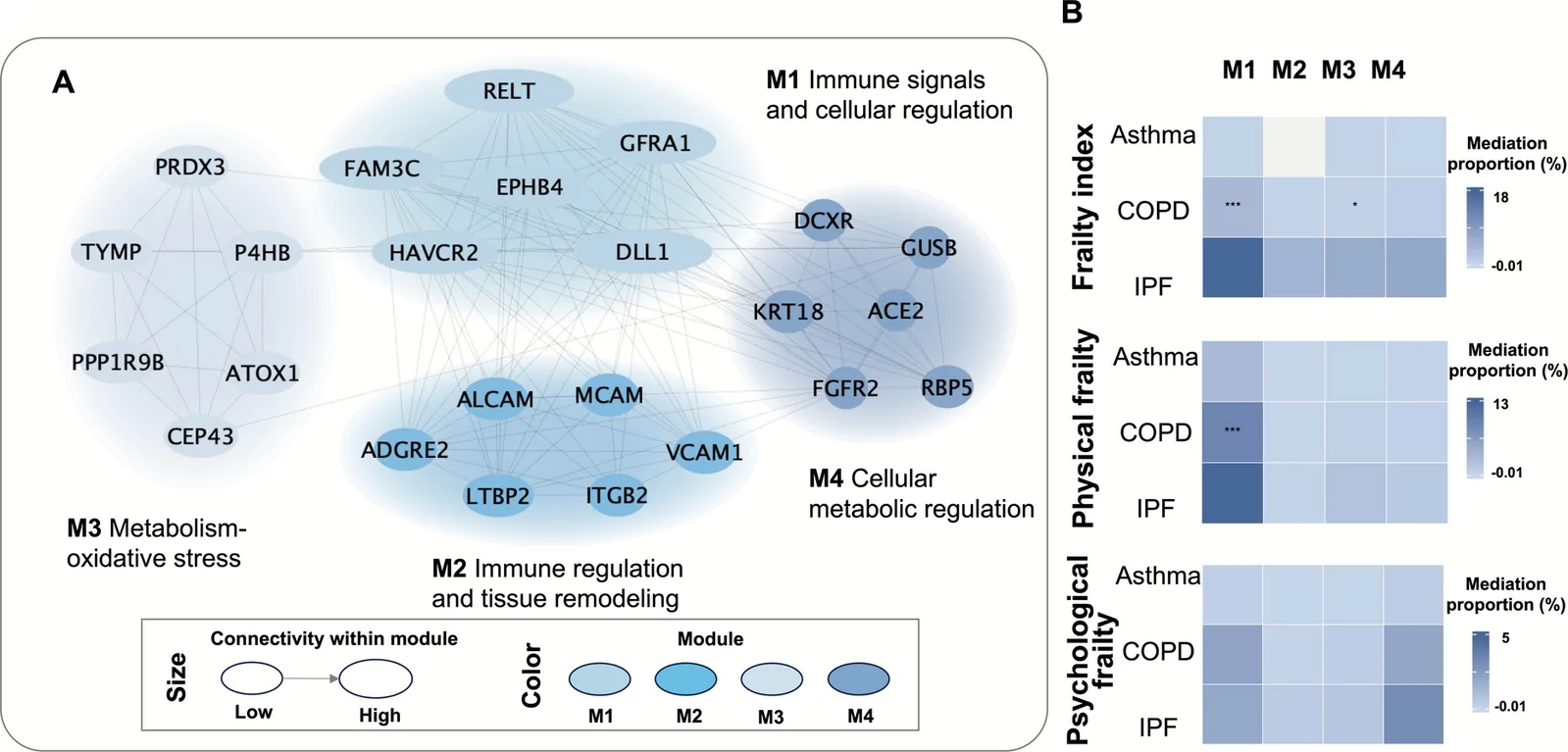
The weighted correlation network analysis of the identified proteins. **A** A protein–protein interaction network for the 6 hub proteins in each co-expression module. The node size reflects the connectivity within modules. **B** heatmaps showing the mediation effects of protein modules on the associations of frailty phenotypes with incident CRDs risk

Mediation analyses revealed that the proteomic signatures played significant intermediary roles in the relationships of frailty index and physical frailty with the incidence risk of COPD, accounting for 26.0% (95% CI: 18.7 to 37.5%, *P* < 0.05) and 60.9% (95% CI: 35.0 to 198.8%, *P* < 0.05) of the total effect, respectively (Fig. 4A). Notably, proteins such as GDF15, WFDC2, and PLAUR were consistently identified as key mediators in these associations (Fig. 4B and Table S36-S38). The mediation results of physical frailty and psychological frailty, as well as lung function are shown in Fig. S4-S5. Four distinct co-expression modules, each containing 45 to 132 proteins, were identified through network analyses (Fig. 5A and Table S39). The functional enrichment analysis identified distinct biological signatures across the four co-expression modules (Fig. S6 and Table S40-S41). Importantly, M1 and M3, which enriched in immune signals, cellular regulation, and metabolism-oxidative stress pathways, were significantly mediated the associations of frailty index and COPD (Fig. 5B and Table S42-S43).

## Discussion

In this study, we systematically characterized circulating proteomic signatures associated with exposure to three frailty phenotypes within a population-based cohort of over 20,000 adults. Our findings delineated triangular associations among frailty phenotypes, plasma protein biomarkers, and the incidence risks of CRDs. Beyond reinforcing the deleterious effects of frailty index and physical frailty on COPD risk, we identified a spectrum of proteins jointly linked to both frailty phenotypes and CRDs, with particular enrichment in immune signals, cellular regulation, and metabolism-oxidative stress pathways.

Previous epidemiological studies demonstrated that frailty, particularly frailty index and physical frailty, were associated with increased risks of adverse respiratory outcomes, including mortality and morbidity in COPD [10, 19, 24]. However, psychological frailty, a key but often overlooked domain, received comparatively little attention in relation to respiratory health outcomes [25]. Moreover, emerging proteomic studies elucidated the molecular underpinnings of frailty and identified related proteins, such as GDF15, CNTN1, WFDC2, and NEFL, involving pathways of inflammation and immune, metabolism, and cellular signaling [26]. Notably, some of these molecular pathways overlap with those involved in the pathogenesis of CRDs, particularly those related to chronic systemic inflammation and immune dysregulation [27]. These findings suggested a potential shared molecular basis linking frailty and CRDs. While there is a lack of integrative studies that systematically evaluate proteomic profiles across multiple frailty domains in relation to incident respiratory disease risk. Our study combined large-scale proteomic profiling with three frailty phenotypes and longitudinal respiratory outcome data, offering novel insights into shared biological mechanisms and potential intervention targets.

Mechanistically, frailty might contribute to the development and progression of CRDs and impairment of lung function through dysregulated immune-inflammatory responses and altered cytokine signaling. Several proteomic markers identified in our study support this hypothesis. For instance, as a stress-responsive cytokine, GDF15 might be upregulated in response to chronic low-grade systemic inflammation and metabolic stress, which are characteristics of frailty [28]. Increased GDF15 could lead to muscle catabolism, impaired mitochondrial function, and immunosenescence. These alterations might exacerbate pulmonary inflammation and promote structural changes in lung tissue, thereby increasing susceptibility to CRDs such as COPD [29]. Additionally, immune dysregulation and epithelial barrier impairment in frail individuals might stimulate overexpression of WFDC2, an innate immunity modulator [26]. Elevated WFDC2 levels were involved in neutrophil recruitment, mucus hypersecretion, and extracellular matrix remodeling in the airway epithelium, which might disrupt normal lung function and contribute to the pathogenesis of asthma, COPD, or other CRDs [30]. Moreover, increased expression of PLAUR, a receptor involved in pericellular proteolysis and immune cell trafficking, may contribute to lung tissue remodeling and sustained inflammation [31]. By enhancing urokinase activity and activating downstream signaling pathways such as MAPK and NF-kappaB, PLAUR may exacerbate airway damage and fibrotic responses, thereby promoting the progression of CRDs [32, 33]. Notably, PLAUR (uPAR) is recognized as a cell-surface marker of senescent cells, with demonstrated elevation in lung and liver fibrosis models [34, 35]. The co-occurrence of high uPAR expression in senescent‐cell enriched fibrotic lung tissue suggests that PLAUR might mediate pathways linking cellular senescence, chronic alveolar injury, and systemic frailty. Given our finding that plasma PLAUR levels correlate strongly with frailty and chronic respiratory disease risk, this mechanistic link reinforces the potential of PLAUR as a biomarker reflecting both local pulmonary pathology and systemic ageing processes.

The observed age-related heterogeneity suggests that frailty-associated proteomic alterations may exert stronger effects on CRD risk during midlife than in later life. One plausible explanation is that frailty manifesting before age 60 represents an accelerated or pathological form of biological aging rather than physiological senescence [36]. Individuals who develop frailty earlier in life are likely to have a higher burden of chronic low-grade inflammation, metabolic stress, and immune dysregulation, which can amplify vulnerability to airway remodeling and pulmonary fibrosis [37]. In contrast, among older adults, frailty becomes more prevalent and heterogeneous, encompassing a mix of biological aging and accumulated comorbidities [38]. This may dilute relative effect sizes even though the overall direction of association remains consistent. Furthermore, respiratory diseases developing in midlife may arise in individuals whose systemic homeostasis has already been disrupted at the molecular level, whereas older individuals may have adapted to chronic physiological decline through compensatory mechanisms [39]. Together, these findings imply that molecular frailty signatures detected in midlife may serve as early indicators of accelerated aging trajectories that predispose individuals to chronic respiratory diseases such as COPD and IPF, highlighting the importance of early detection and intervention strategies targeting systemic dysregulation before overt disease onset.

From a public health and clinical standpoint, our results underscore the potential of frailty, not only as a clinical syndrome but also as an early risk indicator for CRDs. Incorporating frailty assessment into routine population health screening may help identify individuals at elevated risks for CRDs, and further research is warranted to evaluate its predictive utility. Moreover, the identification of specific proteomic mediators highlights a promising biological axis through which frailty may accelerate lung function decline and elevate the risks of CRDs development. This molecular insight supports a shift toward precision public health preventive approaches that leverage biomarkers and informed risk stratification. Hence, integrating frailty and its proteomic profiles into risk assessment frameworks may inform tailored prevention strategies for vulnerable groups. In addition, the study suggests that targeting proteomic pathways associated with frailty may hold potential for delaying or preventing the onset of CRDs, which lays the groundwork for exploring novel therapeutic targets of the implicated proteins [40]. The findings not only deepen our understanding of the complex biological interplay between frailty and chronic respiratory diseases but also pave the way for precision medicine approaches that enable earlier risk stratification and improved long-term outcomes in high-risk populations. Collectively, these results advocate for the expansion of interdisciplinary frameworks bridging clinical geriatrics, molecular epidemiology, and health policy, in alignment with global public health priorities toward aging-friendly and resilient health systems.

Despite the novel contributions of our study, several limitations warrant consideration. First, although the adapted frailty measurements have been previously validated, frailty phenotypes in this study were mostly assessed based on self-reported data, which may be subject to recall bias. Second, while we identified proteomic signatures and potential mediating proteins linking frailty to CRDs and lung function, the observational nature of this study makes it impossible to infer the causality of the results. Experimental validation through in vitro and in vivo models is needed to substantiate the biological pathways implicated in these associations. Third, although elastic net regression allowed the construction of proteomic signatures with robust predictive performance, it relies on the assumption of linearity and does not capture potential nonlinear interactions among proteins. Future research should consider incorporating larger panels of proteins and applying advanced machine learning algorithms to improve model performance and biological interpretability. Fourth, residual confounding, particularly from unmeasured or imperfectly measured socioeconomic factors, cannot be entirely excluded. However, we conducted sensitivity analyses using alternative socioeconomic indicators (e.g., socioeconomic status [SES]) to mitigate this concern. Fifth, although sensitivity analyses were conducted to mitigate potential reverse causation, we acknowledge that residual subclinical respiratory impairment cannot be entirely excluded, and future studies incorporating repeated lung-function assessments and longitudinal frailty trajectories are warranted to clarify temporal ordering. Lastly, the limited ethnic diversity within the UK Biobank restricts the generalizability of our findings. Further replication in more ethnically diverse populations is essential to ensure the broader applicability of the results.

In summary, the present study revealed that frailty phenotypes were associated with increased risks of CRDs, with part of the associations mediated by frailty-related proteins, particularly those enriched in cytokine signaling and immune-inflammatory pathways. The study demonstrated the clinical and biological relevance of frailty as an integrative construct, which could reflect systemic vulnerability for CRDs beyond traditional risk factors. Besides, the findings underscored the potential role of proteomic dysregulation as a biological conduit linking frail stressors to pulmonary dysfunction. Combining frailty assessment with proteomic profiling may help improve our understanding of CRD high-risk populations and the biological mechanisms linking frailty to respiratory dysfunction, particularly among older adults exposed to cumulative environmental and biological stressors.

## Supplementary Information

Below is the link to the electronic supplementary material.

ESM 1 (DOCX 6.43 MB)

ESM 2 (XLSX 4.56 MB)

## Data Availability

The data used in the present study are available from the UK Biobank with restrictions applied. Access to the UK Biobank data can be requested through a standard protocol (https://www.ukbiobank.ac.uk/register-apply/).

## Abbreviations

BMI: Body mass index
CI: Confidence interval
COPD: Chronic obstructive pulmonary disease
CRD: Chronic respiratory diseases
ERS: European Respiratory Society
FDR: False discovery rate
HR: Hazard ratios
ICD: International classification of diseases
IMD: Index of multiple deprivation
IPF: Idiopathic pulmonary fibrosis
SD: Standard deviation
WGCNA: Weighted correlation network analysis
WHR: Waist-to-hip ratio
