# Supplementary material for: Plasma proteomic biomarkers as mediators for the associations between frailty phenotypes and chronic respiratory diseases
Source: GeroScience. 2026 Feb 4;48(6):8285–98. doi: 10.1007/s11357-026-02099-w (PMC13638941; doi:10.1007/s11357-026-02099-w)
Supplement: Supplementary file 1 [file 11357_2026_2099_MOESM1_ESM.docx]

**Plasma proteomic biomarkers as mediators for the association between frailty phenotypes and chronic respiratory diseases**

[eMethods	2](#_Toc214438351)

[Figure S1. Bar plots of the top 20 enriched GO terms and KEGG pathways for identified proteins in elastic net analysis	5](#_Toc214438352)

[Figure S2. Associations between individual plasma proteins and lung function	6](#_Toc214438353)

[Figure S3. The Venn diagrams and mediation analysis of proteomic signatures and individual proteins on the relationship between frailty phenotypes and CRDs	7](#_Toc214438354)

[Figure S4. Associations of frailty phenotypes and proteomic signatures with lung function.	8](#_Toc214438355)

[Figure S5. Mediation analysis of proteomic signatures and individual proteins on the relationship between frailty phenotypes and lung function	9](#_Toc214438356)

[Figure S6. Bar plots of the top 20 enriched GO terms and KEGG pathways for proteins in identified co-expression modules.	10](#_Toc214438357)

[Table S1. Definition of frailty index	11](#_Toc214438358)

[Table S2. Definition of physical frailty	14](#_Toc214438359)

[Table S3. Definition of psychological frailty	15](#_Toc214438360)

[Table S4. Definition of respiratory diseases and lung function	16](#_Toc214438361)

[Table S6. Definition of baseline characteristics, healthy lifestyle behavior, and other environment variables	17](#_Toc214438362)

[Table S7. Definitions of high-risk occupations for respiratory diseases	19](#_Toc214438363)

[Table S8. Definitions of high-risk occupations for environmental factors	20](#_Toc214438364)

[Table S9. Summary of model performance metrics	21](#_Toc214438365)

[Table S10. Baseline characteristics of the participants in the two groups	22](#_Toc214438366)

[Table S11. Baseline characteristics of the participants in the three groups	24](#_Toc214438367)

[Table S12. Baseline characteristics of populations in training and testing sets	26](#_Toc214438368)

[Table S23. HR (95% CI) of frailty and related proteomic signatures with asthma	27](#_Toc214438369)

[Table S24. HR (95% CI) of frailty and related proteomic signatures with COPD	28](#_Toc214438370)

[Table S25. HR (95% CI) of frailty and related proteomic signatures with IPF	29](#_Toc214438371)

[Table S26. HR (95% CI) of frailty and related proteomic signatures with FEV_1_	30](#_Toc214438372)

[Table S27. HR (95% CI) of frailty and related proteomic signatures with FVC	31](#_Toc214438373)

[Table S28. HR (95% CI) of frailty and related proteomic signatures with FEV_1_/FVC	32](#_Toc214438374)

[Table S29. HR (95% CI) of frailty and related proteomic signatures with asthma by age	33](#_Toc214438375)

[Table S30. HR (95% CI) of frailty and related proteomic signatures with COPD by age	35](#_Toc214438376)

[Table S31. HR (95% CI) of frailty and related proteomic signatures with IPF by age	37](#_Toc214438377)

[Table S32. HR (95% CI) of frailty and related proteomic signatures with asthma by sex	39](#_Toc214438378)

[Table S33. HR (95% CI) of frailty and related proteomic signatures with COPD by sex	41](#_Toc214438379)

[Table S34. HR (95% CI) of frailty and related proteomic signatures with IPF by sex	43](#_Toc214438380)

[Table S35. Sensitivity analysis of main associations	45](#_Toc214438381)

# eMethods

**Frailty assessment**

The frailty index was calculated as the proportion of health deficits present in an individual, based on a predefined list of 49 variables related to physical health, chronic conditions, functional limitations, and psychological status. This approach has been previously developed and validated within the UK Biobank [1]. Specifically, we computed the ratio of present deficits to total possible deficits for those missing fewer than 10 items, yielding a score between 0 and 1. Participants were then classified into three categories: non-frail (<0.12), prefrail (0.12–0.24), and frail (>0.24) [2]. Physical frailty mainly focused on the decline of physiological functions and was operationalized based on five components, including weight loss, slowness (gait speed), weakness (hand grip), exhaustion, and low physical activity, which was put forward by Fried and colleagues and then validated in the UK Biobank [3, 4]. Psychological frailty, based on the Tilburg Frailty Indicator, was defined as feelings of depression (3 items), anxiety (2 items), and declines in coping ability (2 items) [5]. Total scores ranged from 0 to 7.

**Secondary outcome ascertainment**

As the secondary outcomes, lung function data were obtained through breath spirometry measurements conducted through standardized procedures with the Vitalograph Pneumotrac 6800 device [6]. Participants were instructed to perform two to three forced expiratory maneuvers within approximately six minutes, each lasting at least six seconds. If the first two attempts were deemed reproducible—defined by a difference of less than 5% in both forced vital capacity (FVC) and forced expiratory volume in one second (FEV_1_)—a third attempt was considered unnecessary by the system. To determine the acceptability of spirometry results, a sample of breathing maneuvers was appraised according to methods described in earlier research [7].

**Proteomic profiling**

The Olink Explore platform employed proximity extension assay technology to generate relative protein quantifications, expressed as log_2_-transformed normalized protein expression values. Comprehensive quality control procedures were applied, including sample filtering, outlier identification, and exclusion of records with QC/assay warnings or suspected sample swaps, following a six-step protocol [8]. A total of 2,923 distinct proteins, including biomarkers such as cardiometabolic, inflammatory, neurological, and oncology, were measured using the Olink Explore 1536 and Explore Expansion panels. Detailed methodologies for sample collection, plasma preparation, proteomic profiling, and quality control procedures have been documented in prior publications [8, 9].

**Covariates**

IMD was applied to measure the relative poverty in small areas, which was made up of seven privative domains, including crime scores, community safety scores, education scores, employment scores, health scores, housing scores, income scores, living environment scores, access to service scores, and physical environment scores [10]. Participants with college or university degree, other professional qualifications, eg: nursing, teaching, and NVQ or HND or HNC or equivalent were defined as high education, and those with a levels/AS levels or equivalent, O levels/GCSEs or equivalent, CSEs or equivalent, and none of the above were defined as low education. WHR was measured by waist/hip measurements and classified as ideal (<0.9 for males and <0.85 for females) and poor. The income, based on the annual average total household income before tax, was categorized into low and high levels based on £31,000 (median)

The variables of healthy lifestyle were calculated using the data collected from a touchscreen questionnaire [11]. The statuses of drinking were classified into current/previous vs. never and smoking was divided into no current smoking (never smokers or ex-smokers) and smoking (current smokers). Five dimensions (early chronotype, 7-8 hours of sleep per day, never/seldom or occasionally insomnia, self-reported no snoring, and never/seldom or occasionally oversleeping) were used to measure healthy sleep, which was classified into no (<4 components) and yes [12]. Regular physical activity was defined as a minimum of 150 minutes of moderate-intensity or 75 minutes of vigorous activity a week or a combination of the two. In terms of healthy diet, seven components were recommended, encompassing more than 3 portions of vegetables per day, 3 portions of fruit per day, 2 portions of fish per week, 3 portions of whole grains per day, and less than 1 serving of processed meat per week, 1.5 portions of raw red meat per week, and 1.5 portions of refined grains per day. Healthy diet was categorized into no (<4 components) and yes.

**Identification of proteomic signatures reflecting air pollution**

Proteins that met the significance threshold (P<0.05) were subjected to the elastic net regression, a hybrid method combining least absolute shrinkage and selection operator (LASSO) regression and Ridge regression. Elastic net regression was implemented in the training set with the mixing parameter (α) set to 0.5 to balance LASSO and Ridge penalties. The optimal penalty parameter (λ) was determined through 10-fold cross-validation to minimize the mean square error. Proteins with non-zero coefficients at the minimum value of λ were retained as the weight associated with each frailty domain.

**Covariate adjustment in regression models**

Three Cox and linear regression models with varying covariate adjustments were constructed, respectively: i) the basic model included adjustments for socio-demographic factors; ii) the multivariable model further adjusted for BMI, WHR, occupational exposure, and lifestyle factors, C-reactive proteins, environmental factors, and prevalent diseases; iii) the mutually adjusted model incorporated both frailty exposure and its related proteomic signature with the adjustments in the multivariable model to assess associations independence. Of note, fasting time, season, and sample age were further adjusted when proteins or proteomic signatures were analyzed in the model.

**Reference**

1.	Williams DM, Jylhävä J, Pedersen NL, Hägg S. A Frailty Index for UK Biobank Participants. *J Gerontol A Biol Sci Med Sci* 2019: 74(4): 582-587.

2.	Zhong Q, Zhou R, Huang YN, Huang RD, Li FR, Chen HW, Wei YF, Liu K, Cao BF, Liao KY, Xu ZY, Wang SA, Wu XB. Frailty and risk of metabolic dysfunction-associated steatotic liver disease and other chronic liver diseases. *J Hepatol* 2025: 82(3): 427-437.

3.	Fried LP, Tangen CM, Walston J, Newman AB, Hirsch C, Gottdiener J, Seeman T, Tracy R, Kop WJ, Burke G, McBurnie MA. Frailty in older adults: evidence for a phenotype. *J Gerontol A Biol Sci Med Sci* 2001: 56(3): M146-156.

4.	Hanlon P, Nicholl BI, Jani BD, Lee D, McQueenie R, Mair FS. Frailty and pre-frailty in middle-aged and older adults and its association with multimorbidity and mortality: a prospective analysis of 493 737 UK Biobank participants. *Lancet Public Health* 2018: 3(7): e323-e332.

5.	Gobbens RJ, van Assen MA, Luijkx KG, Wijnen-Sponselee MT, Schols JM. The Tilburg Frailty Indicator: psychometric properties. *J Am Med Dir Assoc* 2010: 11(5): 344-355.

6.	Shrine N, Guyatt AL, Erzurumluoglu AM, Jackson VE, Hobbs BD, Melbourne CA, Batini C, Fawcett KA, Song K, Sakornsakolpat P, Li X, Boxall R, Reeve NF, Obeidat M, Zhao JH, Wielscher M, Weiss S, Kentistou KA, Cook JP, Sun BB, Zhou J, Hui J, Karrasch S, Imboden M, Harris SE, Marten J, Enroth S, Kerr SM, Surakka I, Vitart V, Lehtimäki T, Allen RJ, Bakke PS, Beaty TH, Bleecker ER, Bossé Y, Brandsma CA, Chen Z, Crapo JD, Danesh J, DeMeo DL, Dudbridge F, Ewert R, Gieger C, Gulsvik A, Hansell AL, Hao K, Hoffman JD, Hokanson JE, Homuth G, Joshi PK, Joubert P, Langenberg C, Li X, Li L, Lin K, Lind L, Locantore N, Luan J, Mahajan A, Maranville JC, Murray A, Nickle DC, Packer R, Parker MM, Paynton ML, Porteous DJ, Prokopenko D, Qiao D, Rawal R, Runz H, Sayers I, Sin DD, Smith BH, Soler Artigas M, Sparrow D, Tal-Singer R, Timmers P, Van den Berge M, Whittaker JC, Woodruff PG, Yerges-Armstrong LM, Troyanskaya OG, Raitakari OT, Kähönen M, Polašek O, Gyllensten U, Rudan I, Deary IJ, Probst-Hensch NM, Schulz H, James AL, Wilson JF, Stubbe B, Zeggini E, Jarvelin MR, Wareham N, Silverman EK, Hayward C, Morris AP, Butterworth AS, Scott RA, Walters RG, Meyers DA, Cho MH, Strachan DP, Hall IP, Tobin MD, Wain LV. New genetic signals for lung function highlight pathways and chronic obstructive pulmonary disease associations across multiple ancestries. *Nat Genet* 2019: 51(3): 481-493.

7.	De Matteis S, Jarvis D, Hutchings S, Darnton A, Fishwick D, Sadhra S, Rushton L, Cullinan P. Occupations associated with COPD risk in the large population-based UK Biobank cohort study. *Occup Environ Med* 2016: 73(6): 378-384.

8.	Sun BB, Chiou J, Traylor M, Benner C, Hsu YH, Richardson TG, Surendran P, Mahajan A, Robins C, Vasquez-Grinnell SG, Hou L, Kvikstad EM, Burren OS, Davitte J, Ferber KL, Gillies CE, Hedman Å K, Hu S, Lin T, Mikkilineni R, Pendergrass RK, Pickering C, Prins B, Baird D, Chen CY, Ward LD, Deaton AM, Welsh S, Willis CM, Lehner N, Arnold M, Wörheide MA, Suhre K, Kastenmüller G, Sethi A, Cule M, Raj A, Burkitt-Gray L, Melamud E, Black MH, Fauman EB, Howson JMM, Kang HM, McCarthy MI, Nioi P, Petrovski S, Scott RA, Smith EN, Szalma S, Waterworth DM, Mitnaul LJ, Szustakowski JD, Gibson BW, Miller MR, Whelan CD. Plasma proteomic associations with genetics and health in the UK Biobank. *Nature* 2023: 622(7982): 329-338.

9.	Eldjarn GH, Ferkingstad E, Lund SH, Helgason H, Magnusson OT, Gunnarsdottir K, Olafsdottir TA, Halldorsson BV, Olason PI, Zink F, Gudjonsson SA, Sveinbjornsson G, Magnusson MI, Helgason A, Oddsson A, Halldorsson GH, Magnusson MK, Saevarsdottir S, Eiriksdottir T, Masson G, Stefansson H, Jonsdottir I, Holm H, Rafnar T, Melsted P, Saemundsdottir J, Norddahl GL, Thorleifsson G, Ulfarsson MO, Gudbjartsson DF, Thorsteinsdottir U, Sulem P, Stefansson K. Large-scale plasma proteomics comparisons through genetics and disease associations. *Nature* 2023: 622(7982): 348-358.

10.	GOV.UK. The English Indices of Deprivation 2019; 2019.

11.	Lourida I, Hannon E, Littlejohns TJ, Langa KM, Hypponen E, Kuzma E, Llewellyn DJ. Association of Lifestyle and Genetic Risk With Incidence of Dementia. *JAMA* 2019: 322(5): 430-437.

12.	Li X, Zhou T, Ma H, Huang T, Gao X, Manson JE, Qi L. Healthy Sleep Patterns and Risk of Incident Arrhythmias. *J Am Coll Cardiol* 2021: 78(12): 1197-1207.

# Figure S1. Bar plots of the top 20 enriched GO terms and KEGG pathways for identified proteins in elastic net analysis

# Figure S2. Associations between individual plasma proteins and lung function

# Figure S3. The Venn diagrams and mediation analysis of proteomic signatures and individual proteins on the relationship between frailty phenotypes and CRDs

# Figure S4. Associations of frailty phenotypes and proteomic signatures with lung function.

# Figure S5. Mediation analysis of proteomic signatures and individual proteins on the relationship between frailty phenotypes and lung function

# Figure S6. Bar plots of the top 20 enriched GO terms and KEGG pathways for proteins in identified co-expression modules.

# Table S1. Definition of frailty index

| **Type of deficit** | **Item** | **Trait** | **Data-Field** | **Categories** | **Coding in FI item** |
| --- | --- | --- | --- | --- | --- |
| Sensory | 1 | Glaucoma | 20002 | no,yes | Categorised 0/1 |
|  | 2 | Cataracts | 20002 | no,yes | Categorised 0/1 |
|  | 3 | Hearing difficulty | 2247 | no, yes, completely deaf | Categorised 0/1 (combined yes/deaf groups as 1) |
| Cranial | 4 | Migraine | 20002 | no,yes | Categorised 0/1 |
|  | 5 | Dental problems | 6149 | ulcers, painful gums, bleeding gums, loose teeth, toothache, dentures | Categorised 0/1 for none vs. any |
| Mental wellbeing | 6 | Self-rated health | 2178 | excellent, good, fair, poor | 0 – excellent; 0.25 – good; 0.5 - fair; 1 – poor |
|  | 7 | Fatigue: frequency of tiredness / lethargy in last two weeks | 2080 | not at all, several days, more than half, nearly every day | 0, 0.25, 0.5, 1, respectively |
|  | 8 | Sleep: experience of sleeplessness/insomnia | 1200 | never/rarely, sometimes, usually | Categorised 0, 0.5, 1, respectively |
|  | 9 | Depressed feelings: frequency in last two weeks | 2050 | not at all, several days, more than half, nearly every day | 0 – not at all, 0.5 – several days, 0.75 - more than half, 1 – nearly every day |
|  | 10 | Self-described nervous personality | 2010 | no, yes | Categorised 0/1 |
|  | 11 | Severe anxiety/ panic attacks | 20002 | no, yes | Categorised 0/1 |
|  | 12 | Common to feel loneliness | 2020 | no, yes | Categorised 0/1 |
|  | 13 | Sense of misery (ever/never) | 1930 | no, yes | Categorised 0/1 |
| Infirmity | 14 | Infirmity: long-standing illness or disability | 2188 | no, yes | Categorised 0/1 |
|  | 15 | Falls in last year | 2296 | categorical: no falls, one fall, more than one | 0, 0.5, 1, respectively |
|  | 16 | Fractures/broken bones in last five years | 2463 | no, yes | Categorised 0/1 |
| Cardiometabolic | 17 | Diabetes | 20002 | no, yes | Categorised 0/1 |
|  | 18 | Myocardial infarction | 20002 | no, yes | Categorised 0/1 |
|  | 19 | Angina | 20002 | no, yes | Categorised 0/1 |
|  | 20 | Stroke | 20002 | no, yes | Categorised 0/1 |
|  | 21 | High blood pressure | 20002 | no, yes | Categorised 0/1 |
|  | 22 | Hypothyroidism | 20002 | no, yes | Categorised 0/1 |
|  | 23 | Deep-vein thrombosis | 20002 | no, yes | Categorised 0/1 |
|  | 24 | High cholesterol | 20002 | no, yes | Categorised 0/1 |
| Respiratory | 25 | Breathing: wheeze in last year | 2316 | no, yes | Categorised 0/1 |
|  | 26 | Pneumonia | 20002 | no, yes | Categorised 0/1 |
|  | 27 | Chronic bronchitis/emphysema | 20002 | no, yes | Categorised 0/1 |
|  | 28 | Asthma | 20002 | no, yes | Categorised 0/1 |
| Musculoskeletal | 29 | Rheumatoid arthriti | 20002 | no, yes | Categorised 0/1 |
|  | 30 | Osteoarthritis | 20002 | no, yes | Categorised 0/1 |
|  | 31 | Gout | 20002 | no, yes | Categorised 0/1 |
|  | 32 | Osteoporosis | 20002 | no, yes | Categorised 0/1 |
| Immunological | 33 | Hayfever, allergic rhinitis or eczema | 20002 | no, yes | Categorised 0/1 |
|  | 34 | Psoriasis | 20002 | no, yes | Categorised 0/1 |
| Cancer | 35 | Any cancer diagnosis | 20001 | no, yes | Categorised 0/1 |
|  | 36 | Multiple cancers diagnosed (number reported) | 134 | O -no cancer or single cancer, 1 -multiple cancers | 0 - no cancer or single cancer, 1 - multiple cancers |
| Pain | 37 | Chest pain | 2335 | no, yes | Categorised 0/1 |
|  | 38 | Head and/or neck pain | 6159 | no, yes (combining responses to pain in head and neck/shoulders) | Categorised 0/1 |
|  | 39 | Back pain | 6159 | no, yes | Categorised 0/1 |
|  | 40 | Stomach/abdominal pain | 6159 | no, yes | Categorised 0/1 |
|  | 41 | Hip pain | 6159 | no, yes | Categorised 0/1 |
|  | 42 | Knee pain | 6159 | no, yes | Categorised 0/1 |
|  | 43 | Whole-body pain | 6159 | no, yes | Categorised 0/1 |
|  | 44 | Facial pain | 20002 | no, yes | Categorised 0/1 |
|  | 45 | Sciatica | 20002 | no, yes | Categorised 0/1 |
| Gastrointestinal | 46 | Gastric reflux | 20002 | no, yes | Categorised 0/1 |
|  | 47 | Hiatus hernia | 20002 | no, yes | Categorised 0/1 |
|  | 48 | Gall stones | 20002 | no, yes | Categorised 0/1 |
|  | 49 | Diverticulitis | 20002 | no, yes | Categorised 0/1 |

# Table S2. Definition of physical frailty

| **Individual’s components** | **Criteria** | **Field IDs** |
| --- | --- | --- |
| **Weight loss** | Self-reported: “Compared with one year ago, has your weight changed?”  Response: yes, lost weight = 1; other = 0;  Do not know/Prefer not to answer = missing data. | 2306 |
| **Exhaustion** | Self-reported: “Over the past two weeks, how often have you felt tired or had little energy?”  Response: more than half the days or nearly every day = 1; other = 0;  Do not know/Prefer not to answer = missing data. | 2080 |
| **Low physical activity** | Quintiles of sex- and age-specific levels of total MET minutes per week derived from IPAQ.  The lowest 20% of total MET minutes per week = 1; other = 0;  No response/ Prefer not to answer = missing data. | 31, 21022, 22037, 22038, 22039 |
| **Slow gait speed** | Self-reported: “How would you describe your usual walking pace?”  Response: slow = 1; other = 0;  Do not know/Prefer not to answer = missing data. | 924 |
| **Low grip strength** | Measured grip strength expressed in kg by sex- and BMI- adjusted cut-off points. Cut-off points: Men If BMI ≤24.0 kg/m^2^ & grip strength ≤29 kg If BMI 24.1 to 26.0 kg/m^2^ & grip strength ≤30 kg If BMI 26.1 to 28.0 kg/m^2^ & grip strength ≤30 kg If BMI >28.0 kg/m^2^ & grip strength ≤32 kg Women If BMI ≤23.0 kg/m^2^ & grip strength ≤17 kg If BMI 23.1 to 26.0 kg/m^2^ & grip strength ≤17.3 kg If BMI 26.1 to 29.0 kg/m^2^ & grip strength ≤18 kg If BMI >29.0 kg/m^2^ & grip strength ≤21 kg  If data on BMI or grip strength is not available = missing data. | 31, 21001, 46, 47 |

# Table S3. Definition of psychological frailty

| **Subdomain** | **Question** | **Category/Field** | **Coding** |
| --- | --- | --- | --- |
| Depressive Symptoms | 1. “Do you ever feel 'just miserable' for no reason?”  2. “Over the past two weeks, how often have you felt down, depressed or hopeless?”  3. “Over the past two weeks, how often have you had little interest or pleasure in doing things?” | 100060/1930, 2050 & 2060 | 1: Yes (1), No (0)  2 & 3: Not at all (0), several days (0.33), more than half (0.67), nearly every day (1) |
| Anxiety Symptoms | 1. “Do you suffer from 'nerves'?”  2. “Over the past two weeks, how often have you felt tense, fidgety or restless?” | 100060/2010 & 2070 | 1: Yes (1), No (0)  2: Not at all (0), several days (0.33), more than half (0.67), nearly every day (1) |
| Coping with Problems | 1. “Are you a worrier?”  2. “Would you call yourself tense or 'highly strung'” | 100060/1980 & 1990 | 1 & 2: Yes (1), No (0) |

# Table S4. Definition of respiratory diseases and lung function

| **Entity** | **Abbr** | **Source** |
| --- | --- | --- |
| **Respiratory diseases** | | |
| Asthma | AD | **New case identification:**  ICD-9: 493  ICD-10: J45, J46 |
| Chronic obstructive pulmonary disease | COPD | **New case identification:**  ICD-9: 492, 496  ICD-10: J43, J44 |
| Idiopathic pulmonary fibrosis | IPF | **New case identification:**  ICD-9: 515, 516  ICD-10: J84 |
| **Lung function** | | |
| Forced expiratory volume in 1-second | FEV1 | **Field code:** 3063 |
| Forced vital capacity | FVC | **Field code:** 3062 |
| FEV1/ FVC | / | **/** |

# Table S6. Definition of baseline characteristics, healthy lifestyle behavior, and other environment variables

| **Characteristics** | **Field code** | **Definition** |
| --- | --- | --- |
| Age | 21022 | UK Biobank baseline characteristics:  This is a derived variable based on date of birth and date of attending an initial assessment center and refers to the age of the participant on the day they attended an Initial Assessment Center, truncated to whole year. |
| Sex | 31 | UK Biobank baseline characteristics:  Acquired from central registry at recruitment, but in some cases updated by the participant. Hence this field may contain a mixture of the sex the National Health Service had recorded for the participant and self-reported sex. |
| Index of multiple deprivation  (IMD) | 26410  26427  26426 | Domains for IMD calculation: crime score (England and Scotland), community safety score (Wales), education score (All), employment score (All), health score (All), housing score (All), income score (All), living environment score (England), access to services score (Scotland and Wales), physical environment score (Wales). |
| Ethnic | 21000 | This is an amalgam of sequential branching questions asked during the initial Assessment Centre visit as part of the touchscreen questionnaire. |
| Income | 738 | Average total household income before tax |
| Body mass index (BMI) | 21001 | BMI value here is constructed from height and weight measured during the initial Assessment Centre visit. |
| Waist-to-hip ratio (WHR) | 48, 49 | WMH was calculated through waist/hip measurement.  **Poor**: ≥0.9 for men; ≥0.85 for women;  **Ideal**: < 0.9 for men; <0.85 for women. |
| Pack years of smoking | 20161 | The general definition of a pack year is the number of cigarettes smoked per day, divided by twenty, multiplied by the number of years of smoking. The number of years of smoking is calculated by subtracting the age of starting smoking from the age smoking was stopped. |
| Never smoking | 20116 | UK Biobank Touchscreen questionnaire at baseline;  This field summarized the current/past smoking status of the participant, divided into current, previous, and never smoker.  **Yes**: never smoking; **No**: current and previous smoking. |
| Moderate drinking | 20117 | Moderate drinking was defined as the average daily intake ≤16 g of pure alcohol (2 units of alcohol) for both men and women. |
| Regular physical activity | 22035 | **Yes**: participants had ≥150 minutes of moderate activity per week or ≥75 minutes of vigorous activity or equivalent combination;  **No**: participants had <150 minutes of moderate activity per week and <75 minutes of vigorous activity. |
| Healthy diet | 1309, 1319  1289, 1299  1329, 1339  1349, 1369  1379, 1389  1438, 1448  1458, 1468 | Seven healthy diet components: i) Fruits: ≥ 3 servings/day; ii) Vegetables: ≥ 3 servings/day; iii) Fish: ≥2 servings/week; iv) Processed meats: ≤ 1 serving/week; v) Unprocessed red meats: ≤ 1.5 servings/week; vi) Whole grains: ≥ 3 servings/day; vii) Refined grains: ≤1.5servings/day.  **Yes**: participants had ≥4 healthy diet components;  **No**: participants had <4 healthy diet components. |
| C-reactive protein | 30710 | CRP was measured by immunoturbidimetric on a Beckman Coulter AU5800. |

# Table S7. Definitions of high-risk occupations for respiratory diseases

| **Entity** | **UKB data field** | **Definition** |
| --- | --- | --- |
| Job code at visit | 20277 | Participants in-service were asked to enumerate their present occupations. The job title is coded according to a tree structure, categorized according to the Standard Occupational Classification 2000. The following occupations have been identified as high-risk occupations for respiratory diseases:   1. Painters and decorators 2. Vehicle spray painters 3. Plastics process operatives 4. Welding trades 5. Carpenters and joiners 6. Cleaners, domestics 7. Laboratory technicians 8. Bakers, flour confectioners 9. Hairdressers, barbers 10. Veterinary nurses and assistants 11. Food, drink and tobacco process operatives 12. Fishing and agriculture related occupations 13. Shelf fillers 14. Elementary personal services occupations 15. Plasterers 16. Artists 17. Textile process operatives 18. Construction operatives 19. Metal working production and maintenance fitters 20. Chemical and related process operatives 21. Quarry workers and related operatives 22. Coal mine operatives |
| Job code - historical | 22617 | Retired participants were asked to enumerate their work history. The job title is coded according to a tree structure, categorized according to the Standard Occupational Classification 2000. The occupations that have been identified as high-risk occupations for respiratory diseases are the same as described above. |

# Table S8. Definitions of high-risk occupations for environmental factors

| **Entity** | **UKB data field** | **Definition** |
| --- | --- | --- |
| Proximity to roadways | 24012 | Proximity to roadways was measured by the inverse distance, which was derived based upon a local road network with traffic intensity greater than 5000 motor vehicles per 24 hours. The local road network is taken from the Ordnance Survey Meridian 2 road network (scale 1:50000, 1 metre accuracy), 2009. |
| Noise pollution (*L_den_*) | 24024 | Noise pollution was classified as low and high exposure according to the *L_den_* of 65 dB. *L_den_*: (day-evening-night equivalent level): A-weighted Leq noise level measured over the 24 hour period with a 10 decibel penality added to the levels between 23:00 and 07:00. |
| Fine particulate matter (PM_2.5_) | / | Annual average concentration of PM_2.5_, fine particulate matter with a size smaller than 2.5μm, was calculated from 2006 to 2010. |
| Nitrogen dioxide (NO_2_) | / | Annual average concentration of NO_2_ was calculated from 2006 to 2010. |

# Table S9. Summary of model performance metrics

|  | AUC | Accuracy | Sensitivity | Specificity | F1-score |
| --- | --- | --- | --- | --- | --- |
| **Training dataset** |  |  |  |  |  |
| Frailty index | 0.746 | 0.703 | 0.478 | 0.850 | 0.561 |
| Physical frailty | 0.741 | 0.678 | 0.729 | 0.618 | 0.712 |
| Psychological frailty | 0.660 | 0.614 | 0.626 | 0.602 | 0.621 |
| **Testing dataset** |  |  |  |  |  |
| Frailty index | 0.727 | 0.698 | 0.468 | 0.844 | 0.546 |
| Physical frailty | 0.737 | 0.678 | 0.73 | 0.617 | 0.710 |
| Psychological frailty | 0.661 | 0.614 | 0.626 | 0.601 | 0.620 |
| **All samples** |  |  |  |  |  |
| Frailty index | 0.740 | 0.701 | 0.475 | 0.848 | 0.557 |
| Physical frailty | 0.740 | 0.678 | 0.730 | 0.618 | 0.711 |
| Psychological frailty | 0.660 | 0.614 | 0.626 | 0.602 | 0.621 |

# Table S10. Baseline characteristics of the participants in the two groups

| **Characteristic** | **All participants**  **(N=22802)** | **Frailty index^a^** | |
| --- | --- | --- | --- |
|  |  | **Non-frail**  **(N=13800)** | **Prefrail and frail**  **(N=9002)** |
| **Age, year** | 57.6 (7.95) | 57.2 (8.04) | 58.3 (7.78) |
| **Female** | 11813 (51.8%) | 7015 (50.8%) | 4798 (53.3%) |
| **IMD^b^** | 16.7 (13.9) | 15.3 (12.8) | 18.9 (15.1) |
| **Low income^c^** | 11791 (51.7%) | 6299 (45.6%) | 5492 (61.0%) |
| **Low education^d^** | 8671 (38.0%) | 4769 (34.6%) | 3902 (43.3%) |
| **White ethnic** | 21708 (95.2%) | 13186 (95.6%) | 8522 (94.7%) |
| **BMI** | 27.6 (4.66) | 27.0 (4.27) | 28.5 (5.06) |
| **Poor WHR^e^** | 11901 (52.2%) | 6626 (48.0%) | 5275 (58.6%) |
| **Occupational exposure^f^** | 2353 (10.3%) | 1383 (10.0%) | 970 (10.8%) |
| **Pack years of smoking** | 21.1 (16.7) | 19.6 (15.6) | 23.5 (18.0) |
| **Healthy lifestyle^g^** |  |  |  |
| Never smoking | 12247 (53.7%) | 7903 (57.3%) | 4344 (48.3%) |
| Moderate drinking | 15255 (66.9%) | 9099 (65.9%) | 6156 (68.4%) |
| Healthy sleep pattern | 11883 (52.1%) | 8332 (60.4%) | 3551 (39.4%) |
| Regular physical activity | 12095 (53.0%) | 7652 (55.4%) | 4443 (49.4%) |
| Healthy diet | 8964 (39.3%) | 5686 (41.2%) | 3278 (36.4%) |
| **Prevalence of comorbidities** |  |  |  |
| Hypertension | 8935 (39.2%) | 4333 (31.4%) | 4602 (51.1%) |
| Diabetes | 1579 (6.92%) | 390 (2.83%) | 1189 (13.2%) |
| **C-reactive protein** | 2.53 (4.19) | 2.25 (3.92) | 2.95 (4.55) |
| **Environmental factors^h^** |  |  |  |
| Noise pollution (*L_den_*), dB | 56.1 (4.28) | 56.0 (4.27) | 56.1 (4.29) |
| Proximity to roadways | 1642 (7.20%) | 971 (7.04%) | 671 (7.45%) |
| PM_2.5_, μg/m^3^ | 10.7 (1.97) | 10.7 (1.95) | 10.7 (1.98) |
| NO_2_, μg/m^3^ | 20.4 (6.44) | 20.2 (6.40) | 20.7 (6.50) |

Abbreviations: IMD, index of multiple deprivation; BMI, body mass index; WHR, waist-hip ratio.

**a** Frailty were categorized into non-frail (≤0.12), prefrail (0.12-0.24), and frail (>0.24). **b** IMD scores offer a detailed view of deprivation. Domains for IMD calculation: crime score, community safety score, education score, employment score, health score, housing score, income score, living environment score, access to services score, physical environment score. **c** Participants with college or university degree, other professional qualifications, and NVQ or HND or HNC or equivalent were defined as high education, and those with A levels/AS levels or equivalent, O levels/GCSE or equivalent, CSE or equivalent, and none of the above were defined as low education. **d** The income was categorized into low and high levels based on £31,000. **e** Waist-to-hip ratio was calculated through waist/hip measurement and was categorized into poor (≥0.9 for men and ≥0.85 for women) and ideal. **f** High-risk occupations for respiratory diseases are shown in Supplementary Table 7. **g** The details of healthy lifestyle could be found in Supplementary Methods. **h** The *L_den_* (weighted average noise sound level for 24 hours, with a penalty of 5 for evening hours and 10 dB for night hours) was a major indicator of noise pollution. The inverse distance was derived based upon a local road network with traffic intensity greater than 5000 motor vehicles per 24 hours. Annual average concentration of PM_2.5_ and NO_2_ were calculated from 2006 to 2010.

# Table S11. Baseline characteristics of the participants in the three groups

| **Characteristic** | **All participants**  **(N=22802)** | **Frailty index^a^** | | |
| --- | --- | --- | --- | --- |
|  |  | **Non-frail**  **(N=13800)** | **Prefrail**  **(N=8637)** | **Frail**  **(N=365)** |
| **Age, year** | 57.6 (7.95) | 57.2 (8.04) | 58.3 (7.78) | 57.9 (7.69) |
| **Female** | 11813 (51.8%) | 7015 (50.8%) | 4613 (53.4%) | 185 (50.7%) |
| **IMD^b^** | 16.7 (13.9) | 15.3 (12.8) | 18.6 (14.9) | 25.2 (18.8) |
| **Low income^c^** | 11791 (51.7%) | 6299 (45.6%) | 5196 (60.2%) | 296 (81.1%) |
| **Low education^d^** | 8671 (38.0%) | 4769 (34.6%) | 3702 (42.9%) | 200 (54.8%) |
| **White ethnic** | 21708 (95.2%) | 13186 (95.6%) | 8181 (94.7%) | 341 (93.4%) |
| **BMI** | 27.6 (4.66) | 27.0 (4.27) | 28.4 (4.97) | 31.5 (6.12) |
| **Poor WHR^e^** | 11901 (52.2%) | 6626 (48.0%) | 5001 (57.9%) | 274 (75.1%) |
| **Occupational exposure^f^** | 2353 (10.3%) | 1383 (10.0%) | 930 (10.8%) | 40 (11.0%) |
| **Pack years of smoking** | 21.1 (16.7) | 19.6 (15.6) | 23.2 (17.8) | 29.7 (21.9) |
| **Healthy lifestyle^g^** |  |  |  |  |
| Never smoking | 12247 (53.7%) | 7903 (57.3%) | 4207 (48.7%) | 137 (37.5%) |
| Moderate drinking | 15255 (66.9%) | 9099 (65.9%) | 5894 (68.2%) | 262 (71.8%) |
| Healthy sleep pattern | 11883 (52.1%) | 8332 (60.4%) | 3490 (40.4%) | 61 (16.7%) |
| Regular physical activity | 12095 (53.0%) | 7652 (55.4%) | 4312 (49.9%) | 131 (35.9%) |
| Healthy diet | 8964 (39.3%) | 5686 (41.2%) | 3156 (36.5%) | 122 (33.4%) |
| **Prevalence of comorbidities** |  |  |  |  |
| Hypertension | 8935 (39.2%) | 4333 (31.4%) | 4323 (50.1%) | 279 (76.4%) |
| Diabetes | 1579 (6.92%) | 390 (2.83%) | 1066 (12.3%) | 123 (33.7%) |
| **C-reactive protein** | 2.53 (4.19) | 2.25 (3.92) | 2.90 (4.49) | 4.19 (5.53) |
| **Environmental factors^h^** |  |  |  |  |
| Noise pollution (*L_den_*), dB | 56.1 (4.28) | 56.0 (4.27) | 56.1 (4.28) | 56.4 (4.57) |
| Proximity to roadways | 1642 (7.20%) | 971 (7.04%) | 637 (7.38%) | 34 (9.32%) |
| PM_2.5_, μg/m^3^ | 10.7 (1.97) | 10.7 (1.95) | 10.7 (1.98) | 10.9 (2.03) |
| NO_2_, μg/m^3^ | 20.4 (6.44) | 20.2 (6.40) | 20.6 (6.49) | 21.6 (6.67) |

Abbreviations: IMD, index of multiple deprivation; BMI, body mass index; WHR, waist-hip ratio.

**a** Frailty were categorized into non-frail (≤0.12), prefrail (0.12-0.24), and frail (>0.24). **b** IMD scores offer a detailed view of deprivation. Domains for IMD calculation: crime score, community safety score, education score, employment score, health score, housing score, income score, living environment score, access to services score, physical environment score. **c** Participants with college or university degree, other professional qualifications, and NVQ or HND or HNC or equivalent were defined as high education, and those with A levels/AS levels or equivalent, O levels/GCSE or equivalent, CSE or equivalent, and none of the above were defined as low education. **d** The income was categorized into low and high levels based on £31,000. **e** Waist-to-hip ratio was calculated through waist/hip measurement and was categorized into poor (≥0.9 for men and ≥0.85 for women) and ideal. **f** High-risk occupations for respiratory diseases are shown in Supplementary Table 7. **g** The details of healthy lifestyle could be found in Supplementary Methods. **h** The *L_den_* (weighted average noise sound level for 24 hours, with a penalty of 5 for evening hours and 10 dB for night hours) was a major indicator of noise pollution. The inverse distance was derived based upon a local road network with traffic intensity greater than 5000 motor vehicles per 24 hours. Annual average concentration of PM_2.5_ and NO_2_ were calculated from 2006 to 2010.

# Table S12. Baseline characteristics of populations in training and testing sets

| **Characteristics** | **All participants**  (N=22802) | **Training set**  (N=15961) | **Testing set**  (N=6841) |
| --- | --- | --- | --- |
| **Frailty phenotypes** |  |  |  |
| Frailty index | 9002 (39.5%) | 6343 (39.7%) | 2659 (38.9%) |
| Physical frailty | 12371 (54.3%) | 8688 (54.4%) | 3683 (53.8%) |
| Psychological frailty | 11514 (50.5%) | 8076 (50.6%) | 3438 (50.3%) |
| **Chronic respiratory diseases** |  |  |  |
| Asthma | 617 (2.71%) | 443 (2.78%) | 174 (2.54%) |
| COPD | 701 (3.07%) | 485 (3.04%) | 216 (3.16%) |
| IPF | 228 (1.00%) | 166 (1.04%) | 62 (0.91%) |
| **Age, year** | 57.6 (7.95) | 57.6 (7.96) | 57.7 (7.93) |
| **Female** | 11813 (51.8%) | 8324 (52.2%) | 3489 (51.0%) |
| **IMD** | 16.7 (13.9) | 16.7 (13.8) | 16.8 (13.9) |
| **Low income** | 11791 (51.7%) | 8244 (51.7%) | 3547 (51.8%) |
| **Low education** | 8671 (38.0%) | 6004 (37.6%) | 2667 (39.0%) |
| **White ethnic** | 21708 (95.2%) | 15201 (95.2%) | 6507 (95.1%) |
| **BMI** | 27.6 (4.66) | 27.6 (4.63) | 27.6 (4.71) |
| **Poor WHR** | 11901 (52.2%) | 8311 (52.1%) | 3590 (52.5%) |
| **Occupational exposure** | 2353 (10.3%) | 1624 (10.2%) | 729 (10.7%) |
| **Pack years of smoking** | 21.1 (16.7) | 21.1 (16.6) | 21.1 (16.9) |
| **Healthy lifestyle** |  |  |  |
| Never smoking | 12247 (53.7%) | 8618 (54.0%) | 3629 (53.0%) |
| Moderate drinking | 15255 (66.9%) | 10691 (67.0%) | 4564 (66.7%) |
| Healthy sleep pattern | 11883 (52.1%) | 8351 (52.3%) | 3532 (51.6%) |
| Regular physical activity | 12095 (53.0%) | 8465 (53.0%) | 3630 (53.1%) |
| Healthy diet | 8964 (39.3%) | 6300 (39.5%) | 2664 (38.9%) |
| **Prevalence of comorbidities** |  |  |  |
| Hypertension | 0.39 (0.49) | 0.39 (0.49) | 0.39 (0.49) |
| Diabetes | 0.07 (0.25) | 0.07 (0.25) | 0.07 (0.26) |
| **C-reactive protein** | 2.53 (4.19) | 2.54 (4.27) | 2.49 (4.02) |
| **Environmental factors** |  |  |  |
| Noise pollution (*L_den_*), dB | 56.1 (4.28) | 56.0 (4.28) | 56.1 (4.27) |
| Proximity to roadways | 1642 (7.20%) | 1143 (7.16%) | 499 (7.29%) |
| PM_2.5_, μg/m^3^ | 10.7 (1.97) | 10.7 (1.96) | 10.7 (1.97) |
| NO_2_, μg/m^3^ | 20.4 (6.44) | 20.4 (6.43) | 20.4 (6.47) |

Abbreviations: COPD, chronic obstructive pulmonary disease; IPF, idiopathic pulmonary fibrosis; IMD, index of multiple deprivation; BMI, body mass index; WHR, waist-hip ratio. Detailed definitions for baseline characteristics, healthy lifestyle factors, prevalent diseases, and environmental factors can be found in Supplementary Table 5-7.

# Table S23. HR (95% CI) of frailty and related proteomic signatures with asthma

| **Models^a^** | **Frailty** | | **Pre-frail related proteomic signature** | **Frail related proteomic signature** |
| --- | --- | --- | --- | --- |
|  | **Pre-frail** | **Frail** |  |  |
| **Frailty index** |  |  |  |  |
| Basic | 1.98 (1.68, 2.34) | 4.01 (2.68, 6.00) | 1.18 (1.09, 1.28) | 1.16 (1.07, 1.26) |
| MV | 1.91 (1.61, 2.27) | 3.61 (2.36, 5.53) | 1.17 (1.08, 1.27) | 1.16 (1.06, 1.26) |
| MV+MA | 1.87 (1.57, 2.22) | 3.33 (2.16, 5.12) | 1.12 (1.03, 1.22) | 1.10 (1.01, 1.20) |
| **Physical frailty** |  |  |  |  |
| Basic | 1.24 (1.05, 1.46) | 1.64 (1.20, 2.24) | 1.28 (1.18, 1.38) | 1.23 (1.13, 1.33) |
| MV | 1.14 (0.95, 1.35) | 1.29 (0.92, 1.81) | 1.19 (1.09, 1.30) | 1.17 (1.07, 1.27) |
| MV+MA | 1.09 (0.91, 1.30) | 1.13 (0.80, 1.60) | 1.18 (1.08, 1.30) | 1.16 (1.06, 1.26) |
| **Psychological frailty** | |  |  |  |
| Basic | 1.25 (1.01, 1.56) | 1.40 (1.14, 1.70) | 0.94 (0.87, 1.02) | 0.97 (0.89, 1.05) |
| MV | 1.26 (1.01, 1.57) | 1.39 (1.13, 1.70) | 0.95 (0.88, 1.03) | 1.04 (0.96, 1.14) |
| MV+MA | 1.28 (1.02, 1.60) | 1.38 (1.12, 1.69) | 0.94 (0.87, 1.02) | 1.03 (0.94, 1.12) |

Abbreviations: MV, multiple variables; MA, mutual adjustment. **a** Three models were applied to estimate the associations. The basic model adjusted for socio-demographic factors. The MV additionally adjusted for BMI, WHR, occupational exposure, and lifestyle factors, C-reactive proteins, environmental factors, and prevalent diseases. The mutually adjusted model (MV+MA) included both frailty phenotypes and its corresponding proteomic signature.

# Table S24. HR (95% CI) of frailty and related proteomic signatures with COPD

| **Models^a^** | **Frailty** | | **Pre-frail related proteomic signature** | **Frail related proteomic signature** |
| --- | --- | --- | --- | --- |
|  | **Pre-frail** | **Frail** |  |  |
| **Frailty index** |  |  |  |  |
| Basic | 2.24 (1.91, 2.63) | 5.41 (3.95, 7.43) | 1.48 (1.40, 1.58) | 1.47 (1.38, 1.56) |
| MV | 1.95 (1.65, 2.30) | 4.52 (3.23, 6.32) | 1.38 (1.29, 1.47) | 1.35 (1.26, 1.45) |
| MV+MA | 1.83 (1.54, 2.16) | 3.56 (2.53, 5.01) | 1.31 (1.23, 1.40) | 1.28 (1.19, 1.38) |
| **Physical frailty** |  |  |  |  |
| Basic | 1.33 (1.13, 1.57) | 2.81 (2.20, 3.59) | 1.91 (1.79, 2.03) | 1.88 (1.77, 2.01) |
| MV | 1.26 (1.06, 1.49) | 2.45 (1.88, 3.21) | 1.74 (1.63, 1.87) | 1.68 (1.56, 1.80) |
| MV+MA | 1.07 (0.90, 1.27) | 1.40 (1.05, 1.85) | 1.70 (1.58, 1.83) | 1.63 (1.52, 1.75) |
| **Psychological frailty** | |  |  |  |
| Basic | 0.90 (0.73, 1.10) | 1.18 (0.99, 1.41) | 0.98 (0.91, 1.06) | 0.92 (0.85, 1.00) |
| MV | 0.86 (0.70, 1.06) | 1.03 (0.86, 1.24) | 1.00 (0.92, 1.08) | 0.99 (0.92, 1.08) |
| MV+MA | 0.86 (0.70, 1.06) | 1.04 (0.87, 1.25) | 1.00 (0.93, 1.08) | 0.99 (0.91, 1.07) |

Abbreviations: MV, multiple variables; MA, mutual adjustment. **a** Three models were applied to estimate the associations. The basic model adjusted for socio-demographic factors. The MV additionally adjusted for BMI, WHR, occupational exposure, and lifestyle factors, C-reactive proteins, environmental factors, and prevalent diseases. The mutually adjusted model (MV+MA) included both frailty phenotypes and its corresponding proteomic signature.

# Table S25. HR (95% CI) of frailty and related proteomic signatures with IPF

| **Models^a^** | **Frailty** | | **Pre-frail related proteomic signature** | **Frail related proteomic signature** |
| --- | --- | --- | --- | --- |
|  | **Pre-frail** | **Frail** |  |  |
| **Frailty index** |  |  |  |  |
| Basic | 1.29 (0.98, 1.68) | 2.65 (1.33, 5.29) | 1.52 (1.37, 1.69) | 1.51 (1.35, 1.69) |
| MV | 1.16 (0.87, 1.54) | 2.08 (1.01, 4.29) | 1.47 (1.32, 1.65) | 1.46 (1.30, 1.65) |
| MV+MA | 1.05 (0.79, 1.41) | 1.53 (0.73, 3.18) | 1.46 (1.30, 1.63) | 1.45 (1.28, 1.64) |
| **Physical frailty** |  |  |  |  |
| Basic | 1.21 (0.92, 1.60) | 2.34 (1.48, 3.69) | 1.77 (1.58, 1.99) | 1.72 (1.52, 1.93) |
| MV | 1.15 (0.86, 1.54) | 1.95 (1.19, 3.20) | 1.68 (1.47, 1.91) | 1.59 (1.40, 1.81) |
| MV+MA | 1.00 (0.74, 1.34) | 1.24 (0.74, 2.07) | 1.66 (1.45, 1.90) | 1.57 (1.37, 1.79) |
| **Psychological frailty** | |  |  |  |
| Basic | 1.14 (0.82, 1.60) | 1.18 (0.85, 1.62) | 0.93 (0.81, 1.07) | 0.95 (0.83, 1.09) |
| MV | 1.13 (0.80, 1.58) | 1.13 (0.82, 1.55) | 0.94 (0.82, 1.07) | 1.02 (0.88, 1.17) |
| MV+MA | 1.15 (0.82, 1.61) | 1.12 (0.81, 1.55) | 0.93 (0.81, 1.07) | 1.01 (0.88, 1.17) |

Abbreviations: MV, multiple variables; MA, mutual adjustment. **a** Three models were applied to estimate the associations. The basic model adjusted for socio-demographic factors. The MV additionally adjusted for BMI, WHR, occupational exposure, and lifestyle factors, C-reactive proteins, environmental factors, and prevalent diseases. The mutually adjusted model (MV+MA) included both frailty phenotypes and its corresponding proteomic signature.

# Table S26. HR (95% CI) of frailty and related proteomic signatures with FEV_1_

| **Models^a^** | **Frailty** | | **Pre-frail related proteomic signature** | **Frail related proteomic signature** |
| --- | --- | --- | --- | --- |
|  | **Pre-frail** | **Frail** |  |  |
| **Frailty index** |  |  |  |  |
| Basic | -0.14 (-0.16, -0.12) | -0.39 (-0.47, -0.32) | -0.09 (-0.10, -0.08) | -0.08 (-0.09, -0.07) |
| MV | -0.08 (-0.10, -0.06) | -0.24 (-0.32, -0.17) | -0.07 (-0.08, -0.06) | -0.06 (-0.07, -0.05) |
| MV+MA | -0.07 (-0.09, -0.05) | -0.20 (-0.28, -0.13) | -0.06 (-0.07, -0.05) | -0.06 (-0.07, -0.04) |
| **Physical frailty** |  |  |  |  |
| Basic | -0.12 (-0.14, -0.10) | -0.35 (-0.39, -0.31) | -0.13 (-0.14, -0.12) | -0.10 (-0.11, -0.09) |
| MV | -0.09 (-0.11, -0.07) | -0.25 (-0.30, -0.21) | -0.09 (-0.10, -0.08) | -0.07 (-0.08, -0.06) |
| MV+MA | -0.07 (-0.09, -0.05) | -0.20 (-0.25, -0.16) | -0.08 (-0.09, -0.07) | -0.06 (-0.07, -0.05) |
| **Psychological frailty** | |  |  |  |
| Basic | 0.02 (-0.01, 0.04) | -0.02 (-0.04, 0.00) | 0.01 (0.00, 0.02) | 0.02 (0.01, 0.03) |
| MV | 0.02 (-0.01, 0.04) | -0.01 (-0.03, 0.01) | 0.01 (0.00, 0.02) | -0.01 (-0.02, 0.00) |
| MV+MA | 0.01 (-0.01, 0.04) | -0.01 (-0.03, 0.01) | 0.01 (0.00, 0.02) | -0.01 (-0.02, 0.00) |

Abbreviations: MV, multiple variables; MA, mutual adjustment. **a** Three models were applied to estimate the associations. The basic model adjusted for socio-demographic factors. The MV additionally adjusted for BMI, WHR, occupational exposure, and lifestyle factors, C-reactive proteins, environmental factors, and prevalent diseases. The mutually adjusted model (MV+MA) included both frailty phenotypes and its corresponding proteomic signature.

# Table S27. HR (95% CI) of frailty and related proteomic signatures with FVC

| **Models^a^** | **Frailty** | | **Pre-frail related proteomic signature** | **Frail related proteomic signature** |
| --- | --- | --- | --- | --- |
|  | **Pre-frail** | **Frail** |  |  |
| **Frailty index** |  |  |  |  |
| Basic | -0.13 (-0.15, -0.11) | -0.39 (-0.46, -0.31) | -0.07 (-0.08, -0.06) | -0.07 (-0.08, -0.06) |
| MV | -0.07 (-0.09, -0.05) | -0.22 (-0.30, -0.15) | -0.05 (-0.06, -0.04) | -0.05 (-0.06, -0.04) |
| MV+MA | -0.07 (-0.09, -0.05) | -0.19 (-0.27, -0.11) | -0.05 (-0.06, -0.04) | -0.04 (-0.05, -0.03) |
| **Physical frailty** |  |  |  |  |
| Basic | -0.13 (-0.15, -0.11) | -0.35 (-0.39, -0.30) | -0.11 (-0.12, -0.10) | -0.08 (-0.09, -0.07) |
| MV | -0.08 (-0.10, -0.06) | -0.22 (-0.27, -0.18) | -0.06 (-0.07, -0.05) | -0.05 (-0.06, -0.03) |
| MV+MA | -0.07 (-0.09, -0.05) | -0.19 (-0.24, -0.15) | -0.05 (-0.06, -0.04) | -0.04 (-0.05, -0.02) |
| **Psychological frailty** | |  |  |  |
| Basic | 0.01 (-0.02, 0.03) | -0.01 (-0.03, 0.01) | 0.01 (0.00, 0.02) | 0.01 (0.00, 0.02) |
| MV | 0.00 (-0.02, 0.03) | -0.01 (-0.03, 0.01) | 0.01 (0.00, 0.02) | -0.02 (-0.03, -0.01) |
| MV+MA | 0.00 (-0.02, 0.03) | 0.00 (-0.03, 0.02) | 0.01 (0.00, 0.02) | -0.01 (-0.02, 0.00) |

Abbreviations: MV, multiple variables; MA, mutual adjustment. **a** Three models were applied to estimate the associations. The basic model adjusted for socio-demographic factors. The MV additionally adjusted for BMI, WHR, occupational exposure, and lifestyle factors, C-reactive proteins, environmental factors, and prevalent diseases. The mutually adjusted model (MV+MA) included both frailty phenotypes and its corresponding proteomic signature.

# Table S28. HR (95% CI) of frailty and related proteomic signatures with FEV_1_/FVC

| **Models^a^** | **Frailty** | | **Pre-frail related proteomic signature** | **Frail related proteomic signature** |
| --- | --- | --- | --- | --- |
|  | **Pre-frail** | **Frail** |  |  |
| **Frailty index** |  |  |  |  |
| Basic | -0.02 (-0.04, 0.01) | -0.01 (-0.11, 0.08) | -0.05 (-0.06, -0.04) | -0.05 (-0.06, -0.03) |
| MV | -0.03 (-0.06, 0.00) | -0.07 (-0.17, 0.02) | -0.04 (-0.06, -0.03) | -0.04 (-0.05, -0.02) |
| MV+MA | -0.02 (-0.05, 0.00) | -0.05 (-0.14, 0.05) | -0.04 (-0.05, -0.03) | -0.04 (-0.05, -0.02) |
| **Physical frailty** |  |  |  |  |
| Basic | 0.02 (-0.01, 0.04) | 0.01 (-0.05, 0.06) | -0.05 (-0.06, -0.03) | -0.05 (-0.07, -0.04) |
| MV | -0.01 (-0.04, 0.01) | -0.07 (-0.13, -0.01) | -0.06 (-0.08, -0.05) | -0.06 (-0.07, -0.04) |
| MV+MA | 0.00 (-0.03, 0.03) | -0.02 (-0.08, 0.04) | -0.06 (-0.08, -0.05) | -0.05 (-0.07, -0.04) |
| **Psychological frailty** | |  |  |  |
| Basic | 0.01 (-0.02, 0.04) | -0.02 (-0.05, 0.01) | 0.00 (-0.01, 0.01) | 0.00 (-0.01, 0.01) |
| MV | 0.02 (-0.01, 0.05) | 0.00 (-0.03, 0.03) | 0.00 (-0.01, 0.01) | 0.02 (0.00, 0.03) |
| MV+MA | 0.02 (-0.01, 0.05) | -0.01 (-0.04, 0.02) | 0.00 (-0.01, 0.01) | 0.02 (0.00, 0.03) |

Abbreviations: MV, multiple variables; MA, mutual adjustment. **a** Three models were applied to estimate the associations. The basic model adjusted for socio-demographic factors. The MV additionally adjusted for BMI, WHR, occupational exposure, and lifestyle factors, C-reactive proteins, environmental factors, and prevalent diseases. The mutually adjusted model (MV+MA) included both frailty phenotypes and its corresponding proteomic signature.

# Table S29. HR (95% CI) of frailty and related proteomic signatures with asthma by age

|  | **Models^a^** | **Age** | | **P for interaction** |
| --- | --- | --- | --- | --- |
|  |  | **<60** | **≥60** |  |
| **Frailty** | **Frailty index** |  |  |  |
|  | Basic | 1.88 (1.57, 2.24) | 2.38 (1.75, 3.23) | 0.969 |
|  | MV | 1.87 (1.56, 2.25) | 2.41 (1.77, 3.28) | 0.876 |
|  | MV+MA | 1.72 (1.43, 2.07) | 2.17 (1.59, 2.96) | 0.897 |
|  | **Physical frailty** |  |  |  |
|  | Basic | 1.21 (1.02, 1.45) | 1.25 (0.92, 1.69) | 0.969 |
|  | MV | 1.21 (1.01, 1.45) | 1.24 (0.92, 1.69) | 0.949 |
|  | MV+MA | 1.09 (0.91, 1.32) | 1.12 (0.82, 1.53) | 0.973 |
|  | **Psychological frailty** |  |  |  |
|  | Basic | 1.23 (1.01, 1.51) | 1.5 (1.08, 2.08) | 0.969 |
|  | MV | 1.2 (0.98, 1.48) | 1.48 (1.07, 2.06) | 0.876 |
|  | MV+MA | 1.2 (0.98, 1.47) | 1.47 (1.06, 2.05) | 0.897 |
| **Frailty signiture** | **Frailty index** |  |  |  |
|  | Basic | 1.27 (1.17, 1.38) | 1.29 (1.13, 1.47) | 0.969 |
|  | MV | 1.32 (1.2, 1.45) | 1.35 (1.18, 1.56) | 0.949 |
|  | MV+MA | 1.21 (1.1, 1.34) | 1.25 (1.08, 1.44) | 0.973 |
|  | **Physical frailty** |  |  |  |
|  | Basic | 1.24 (1.13, 1.35) | 1.25 (1.08, 1.44) | 0.969 |
|  | MV | 1.24 (1.13, 1.36) | 1.26 (1.09, 1.46) | 0.949 |
|  | MV+MA | 1.22 (1.11, 1.35) | 1.24 (1.07, 1.45) | 0.973 |
|  | **Psychological frailty** |  |  |  |
|  | Basic | 1.02 (0.93, 1.11) | 1.02 (0.88, 1.18) | 0.969 |
|  | MV | 1.02 (0.94, 1.12) | 1.03 (0.89, 1.19) | 0.949 |
|  | MV+MA | 1.01 (0.92, 1.10) | 1.01 (0.87, 1.17) | 0.973 |

Abbreviations: MV, multiple variables; MA, mutual adjustment. **a** Three models were applied to estimate the associations. The basic model adjusted for socio-demographic factors. The MV additionally adjusted for BMI, WHR, occupational exposure, and lifestyle factors, C-reactive proteins, environmental factors, and prevalent diseases. The mutually adjusted model (MV+MA) included both frailty phenotypes and its corresponding proteomic signature.

# Table S30. HR (95% CI) of frailty and related proteomic signatures with COPD by age

|  | **Models^a^** | **Age** | | **P for interaction** |
| --- | --- | --- | --- | --- |
|  |  | **<60** | **≥60** |  |
| **Frailty** | **Frailty index** |  |  |  |
|  | Basic | 2.48 (2.04, 3.02) | 2.06 (1.62, 2.62) | 0.322 |
|  | MV | 2.03 (1.66, 2.49) | 1.72 (1.35, 2.20) | 0.426 |
|  | MV+MA | 1.57 (1.27, 1.92) | 1.32 (1.03, 1.69) | 0.413 |
|  | **Physical frailty** |  |  |  |
|  | Basic | 1.34 (1.11, 1.62) | 1.66 (1.29, 2.13) | 0.322 |
|  | MV | 1.25 (1.03, 1.52) | 1.55 (1.20, 2.00) | 0.368 |
|  | MV+MA | 0.95 (0.78, 1.16) | 1.18 (0.91, 1.53) | 0.413 |
|  | **Psychological frailty** |  |  |  |
|  | Basic | 0.95 (0.77, 1.16) | 1.13 (0.89, 1.45) | 0.322 |
|  | MV | 0.84 (0.68, 1.02) | 1.04 (0.81, 1.32) | 0.368 |
|  | MV+MA | 0.89 (0.72, 1.09) | 1.08 (0.84, 1.38) | 0.413 |
| **Frailty signiture** | **Frailty index** |  |  |  |
|  | Basic | 1.92 (1.78, 2.07) | 1.58 (1.43, 1.74) | 0.006 |
|  | MV | 1.85 (1.70, 2.01) | 1.56 (1.40, 1.74) | 0.048 |
|  | MV+MA | 1.77 (1.62, 1.93) | 1.49 (1.33, 1.66) | 0.036 |
|  | **Physical frailty** |  |  |  |
|  | Basic | 1.85 (1.71, 2.01) | 1.71 (1.55, 1.89) | 0.322 |
|  | MV | 1.72 (1.57, 1.88) | 1.62 (1.46, 1.81) | 0.468 |
|  | MV+MA | 1.71 (1.56, 1.88) | 1.62 (1.45, 1.81) | 0.473 |
|  | **Psychological frailty** |  |  |  |
|  | Basic | 0.79 (0.72, 0.87) | 0.76 (0.67, 0.85) | 0.525 |
|  | MV | 0.86 (0.79, 0.95) | 0.83 (0.73, 0.93) | 0.536 |
|  | MV+MA | 0.87 (0.79, 0.95) | 0.83 (0.74, 0.93) | 0.528 |

Abbreviations: COPD, chronic obstructive pulmonary disease; MV, multiple variables; MA, mutual adjustment. **a** Three models were applied to estimate the associations. The basic model adjusted for socio-demographic factors. The MV additionally adjusted for BMI, WHR, occupational exposure, and lifestyle factors, C-reactive proteins, environmental factors, and prevalent diseases. The mutually adjusted model (MV+MA) included both frailty phenotypes and its corresponding proteomic signature.

# Table S31. HR (95% CI) of frailty and related proteomic signatures with IPF by age

|  | **Models^a^** | **Age** | | **P for interaction** |
| --- | --- | --- | --- | --- |
|  |  | **<60** | **≥60** |  |
| **Frailty** | **Frailty index** |  |  |  |
|  | Basic | 1.77 (1.27, 2.49) | 1.07 (0.75, 1.54) | 0.088 |
|  | MV | 1.55 (1.09, 2.19) | 0.94 (0.65, 1.37) | 0.094 |
|  | MV+MA | 1.14 (0.80, 1.63) | 0.67 (0.45, 0.98) | 0.062 |
|  | **Physical frailty** |  |  |  |
|  | Basic | 1.51 (1.07, 2.11) | 1.21 (0.83, 1.75) | 0.384 |
|  | MV | 1.39 (0.98, 1.97) | 1.12 (0.76, 1.64) | 0.397 |
|  | MV+MA | 1.12 (0.78, 1.60) | 0.89 (0.60, 1.31) | 0.371 |
|  | **Psychological frailty** |  |  |  |
|  | Basic | 1.29 (0.88, 1.90) | 0.94 (0.65, 1.36) | 0.358 |
|  | MV | 1.22 (0.83, 1.80) | 0.90 (0.62, 1.31) | 0.382 |
|  | MV+MA | 1.27 (0.86, 1.87) | 0.93 (0.64, 1.35) | 0.371 |
| **Frailty signiture** | **Frailty index** |  |  |  |
|  | Basic | 2.05 (1.80, 2.34) | 1.49 (1.27, 1.74) | 0.006 |
|  | MV | 2.10 (1.82, 2.43) | 1.52 (1.29, 1.80) | 0.012 |
|  | MV+MA | 2.13 (1.83, 2.47) | 1.55 (1.30, 1.84) | 0.012 |
|  | **Physical frailty** |  |  |  |
|  | Basic | 1.90 (1.65, 2.20) | 1.45 (1.23, 1.72) | 0.039 |
|  | MV | 1.79 (1.53, 2.10) | 1.37 (1.15, 1.64) | 0.045 |
|  | MV+MA | 1.79 (1.52, 2.10) | 1.37 (1.14, 1.65) | 0.045 |
|  | **Psychological frailty** |  |  |  |
|  | Basic | 0.90 (0.76, 1.07) | 0.80 (0.67, 0.96) | 0.384 |
|  | MV | 0.95 (0.8, 1.12) | 0.85 (0.71, 1.02) | 0.397 |
|  | MV+MA | 0.94 (0.8, 1.12) | 0.84 (0.70, 1.02) | 0.371 |

Abbreviations: IPF, idiopathic pulmonary fibrosis; MV, multiple variables; MA, mutual adjustment. **a** Three models were applied to estimate the associations. The basic model adjusted for socio-demographic factors. The MV additionally adjusted for BMI, WHR, occupational exposure, and lifestyle factors, C-reactive proteins, environmental factors, and prevalent diseases. The mutually adjusted model (MV+MA) included both frailty phenotypes and its corresponding proteomic signature.

# Table S32. HR (95% CI) of frailty and related proteomic signatures with asthma by sex

|  | **Models^a^** | **Sex** | | **P for interaction** |
| --- | --- | --- | --- | --- |
|  |  | **Female** | **Male** |  |
| **Frailty** | **Frailty index** |  |  |  |
|  | Basic | 2.00 (1.63, 2.44) | 1.98 (1.57, 2.48) | 0.942 |
|  | MV | 2.00 (1.63, 2.46) | 2.01 (1.59, 2.54) | 0.980 |
|  | MV+MA | 1.84 (1.49, 2.27) | 1.84 (1.45, 2.34) | 0.989 |
|  | **Physical frailty** |  |  |  |
|  | Basic | 1.23 (1.01, 1.51) | 1.18 (0.94, 1.48) | 0.942 |
|  | MV | 1.22 (0.99, 1.51) | 1.18 (0.94, 1.49) | 0.980 |
|  | MV+MA | 1.11 (0.90, 1.38) | 1.08 (0.85, 1.37) | 0.989 |
|  | **Psychological frailty** |  |  |  |
|  | Basic | 1.19 (0.94, 1.52) | 1.47 (1.15, 1.88) | 0.708 |
|  | MV | 1.17 (0.91, 1.49) | 1.44 (1.12, 1.84) | 0.706 |
|  | MV+MA | 1.16 (0.91, 1.49) | 1.43 (1.12, 1.84) | 0.732 |
| **Frailty signiture** | **Frailty index** |  |  |  |
|  | Basic | 1.31 (1.19, 1.45) | 1.21 (1.09, 1.34) | 0.708 |
|  | MV | 1.35 (1.22, 1.50) | 1.27 (1.13, 1.42) | 0.706 |
|  | MV+MA | 1.24 (1.11, 1.38) | 1.16 (1.03, 1.31) | 0.732 |
|  | **Physical frailty** |  |  |  |
|  | Basic | 1.21 (1.09, 1.34) | 1.23 (1.1, 1.38) | 0.942 |
|  | MV | 1.22 (1.09, 1.35) | 1.25 (1.11, 1.40) | 0.980 |
|  | MV+MA | 1.20 (1.07, 1.34) | 1.23 (1.09, 1.39) | 0.989 |
|  | **Psychological frailty** |  |  |  |
|  | Basic | 1.04 (0.94, 1.15) | 0.97 (0.86, 1.09) | 0.710 |
|  | MV | 1.05 (0.95, 1.16) | 0.98 (0.87, 1.10) | 0.706 |
|  | MV+MA | 1.04 (0.94, 1.15) | 0.96 (0.85, 1.08) | 0.732 |

Abbreviations: MV, multiple variables; MA, mutual adjustment. **a** Three models were applied to estimate the associations. The basic model adjusted for socio-demographic factors. The MV additionally adjusted for BMI, WHR, occupational exposure, and lifestyle factors, C-reactive proteins, environmental factors, and prevalent diseases. The mutually adjusted model (MV+MA) included both frailty phenotypes and its corresponding proteomic signature.

# Table S33. HR (95% CI) of frailty and related proteomic signatures with COPD by sex

|  | **Models^a^** | **Sex** | | **P for interaction** |
| --- | --- | --- | --- | --- |
|  |  | **Female** | **Male** |  |
| **Frailty** | **Frailty index** |  |  |  |
|  | Basic | 2.27 (1.79, 2.88) | 2.33 (1.92, 2.84) | 0.984 |
|  | MV | 1.98 (1.56, 2.52) | 1.92 (1.57, 2.35) | 0.860 |
|  | MV+MA | 1.56 (1.22, 1.99) | 1.49 (1.21, 1.84) | 0.860 |
|  | **Physical frailty** |  |  |  |
|  | Basic | 1.35 (1.06, 1.71) | 1.45 (1.20, 1.76) | 0.927 |
|  | MV | 1.26 (0.99, 1.6) | 1.37 (1.12, 1.67) | 0.860 |
|  | MV+MA | 0.98 (0.76, 1.25) | 1.07 (0.88, 1.31) | 0.808 |
|  | **Psychological frailty** |  |  |  |
|  | Basic | 0.88 (0.68, 1.15) | 1.18 (0.97, 1.43) | 0.276 |
|  | MV | 0.79 (0.61, 1.02) | 1.05 (0.86, 1.28) | 0.288 |
|  | MV+MA | 0.83 (0.64, 1.08) | 1.11 (0.91, 1.35) | 0.285 |
| **Frailty signiture** | **Frailty index** |  |  |  |
|  | Basic | 1.83 (1.67, 2.02) | 1.66 (1.54, 1.79) | 0.276 |
|  | MV | 1.79 (1.62, 1.99) | 1.62 (1.48, 1.77) | 0.288 |
|  | MV+MA | 1.70 (1.53, 1.90) | 1.53 (1.40, 1.68) | 0.285 |
|  | **Physical frailty** |  |  |  |
|  | Basic | 1.63 (1.47, 1.81) | 1.8 (1.65, 1.95) | 0.276 |
|  | MV | 1.56 (1.4, 1.74) | 1.67 (1.53, 1.83) | 0.578 |
|  | MV+MA | 1.55 (1.39, 1.73) | 1.67 (1.52, 1.83) | 0.566 |
|  | **Psychological frailty** |  |  |  |
|  | Basic | 0.78 (0.7, 0.88) | 0.78 (0.71, 0.86) | 0.984 |
|  | MV | 0.86 (0.77, 0.96) | 0.85 (0.77, 0.93) | 0.860 |
|  | MV+MA | 0.86 (0.76, 0.96) | 0.85 (0.77, 0.93) | 0.860 |

Abbreviations: COPD, chronic obstructive pulmonary disease; MV, multiple variables; MA, mutual adjustment. **a** Three models were applied to estimate the associations. The basic model adjusted for socio-demographic factors. The MV additionally adjusted for BMI, WHR, occupational exposure, and lifestyle factors, C-reactive proteins, environmental factors, and prevalent diseases. The mutually adjusted model (MV+MA) included both frailty phenotypes and its corresponding proteomic signature.

# Table S34. HR (95% CI) of frailty and related proteomic signatures with IPF by sex

|  | **Models^a^** | **Sex** | | **P for interaction** |
| --- | --- | --- | --- | --- |
|  |  | **Female** | **Male** |  |
| **Frailty** | **Frailty index** |  |  |  |
|  | Basic | 1.55 (1.04, 2.31) | 1.32 (0.96, 1.80) | 0.637 |
|  | MV | 1.45 (0.96, 2.18) | 1.16 (0.83, 1.61) | 0.593 |
|  | MV+MA | 1.07 (0.71, 1.63) | 0.85 (0.61, 1.19) | 0.589 |
|  | **Physical frailty** |  |  |  |
|  | Basic | 1.52 (0.99, 2.32) | 1.24 (0.91, 1.70) | 0.637 |
|  | MV | 1.43 (0.93, 2.19) | 1.15 (0.83, 1.59) | 0.593 |
|  | MV+MA | 1.18 (0.76, 1.83) | 0.95 (0.68, 1.32) | 0.589 |
|  | **Psychological frailty** |  |  |  |
|  | Basic | 0.85 (0.55, 1.32) | 1.35 (0.97, 1.87) | 0.303 |
|  | MV | 0.81 (0.52, 1.27) | 1.28 (0.92, 1.78) | 0.324 |
|  | MV+MA | 0.84 (0.54, 1.32) | 1.33 (0.95, 1.85) | 0.315 |
| **Frailty signiture** | **Frailty index** |  |  |  |
|  | Basic | 1.75 (1.47, 2.08) | 1.68 (1.48, 1.91) | 0.699 |
|  | MV | 1.79 (1.49, 2.15) | 1.72 (1.49, 1.99) | 0.706 |
|  | MV+MA | 1.81 (1.50, 2.18) | 1.74 (1.50, 2.02) | 0.704 |
|  | **Physical frailty** |  |  |  |
|  | Basic | 1.51 (1.25, 1.82) | 1.66 (1.44, 1.91) | 0.637 |
|  | MV | 1.44 (1.19, 1.75) | 1.56 (1.33, 1.82) | 0.593 |
|  | MV+MA | 1.43 (1.18, 1.75) | 1.55 (1.33, 1.82) | 0.589 |
|  | **Psychological frailty** |  |  |  |
|  | Basic | 1.01 (0.82, 1.24) | 0.77 (0.65, 0.91) | 0.258 |
|  | MV | 1.06 (0.86, 1.29) | 0.82 (0.69, 0.96) | 0.300 |
|  | MV+MA | 1.05 (0.86, 1.28) | 0.81 (0.68, 0.95) | 0.282 |

Abbreviations: IPF, idiopathic pulmonary fibrosis; MV, multiple variables; MA, mutual adjustment. **a** Three models were applied to estimate the associations. The basic model adjusted for socio-demographic factors. The MV additionally adjusted for BMI, WHR, occupational exposure, and lifestyle factors, C-reactive proteins, environmental factors, and prevalent diseases. The mutually adjusted model (MV+MA) included both frailty phenotypes and its corresponding proteomic signature.

# Table S35. Sensitivity analysis of main associations

| **Sensitivity analysis^a^** | **Models^b^** | | |
| --- | --- | --- | --- |
|  | Basic  HR (95% CI) | MV  HR (95% CI) | MV+MA  HR (95% CI) |
| **Sensitivity analysis 1 for asthma** |  |  |  |
| Frailty index | 1.98 (1.68, 2.34) | 1.88 (1.58, 2.24) | 1.78 (1.49, 2.13) |
| Physical frailty | 1.27 (1.08, 1.51) | 1.15 (0.97, 1.38) | 1.10 (0.92, 1.32) |
| Psychological frailty | 1.28 (1.08, 1.51) | 1.25 (1.06, 1.48) | 1.22 (1.03, 1.44) |
| Frailty index-proteomic signature | 1.26 (1.16, 1.37) | 1.23 (1.12, 1.35) | 1.14 (1.03, 1.26) |
| Physical frailty -proteomic signature | 1.24 (1.13, 1.35) | 1.15 (1.04, 1.27) | 1.13 (1.02, 1.25) |
| Psychological frailty-proteomic signature | 1.06 (0.98, 1.16) | 1.11 (1.02, 1.21) | 1.10 (1.00, 1.20) |
| **Sensitivity analysis 1 for COPD** |  |  |  |
| Frailty index | 2.37 (2.02, 2.77) | 2.03 (1.71, 2.40) | 1.62 (1.36, 1.93) |
| Physical frailty | 1.48 (1.26, 1.74) | 1.34 (1.13, 1.58) | 1.08 (0.91, 1.29) |
| Psychological frailty | 1.27 (1.09, 1.49) | 1.14 (0.98, 1.33) | 1.13 (0.97, 1.32) |
| Frailty index-proteomic signature | 1.70 (1.59, 1.82) | 1.66 (1.54, 1.80) | 1.57 (1.44, 1.70) |
| Physical frailty -proteomic signature | 1.69 (1.58, 1.81) | 1.63 (1.50, 1.77) | 1.62 (1.49, 1.76) |
| Psychological frailty-proteomic signature | 1.02 (0.94, 1.10) | 1.04 (0.96, 1.12) | 1.03 (0.95, 1.12) |
| **Sensitivity analysis 1 for IPF** |  |  |  |
| Frailty index | 1.35 (1.03, 1.76) | 1.19 (0.90, 1.59) | 0.91 (0.67, 1.22) |
| Physical frailty | 1.39 (1.05, 1.83) | 1.25 (0.94, 1.68) | 1.05 (0.78, 1.42) |
| Psychological frailty | 1.23 (0.94, 1.61) | 1.18 (0.90, 1.54) | 1.19 (0.90, 1.57) |
| Frailty index-proteomic signature | 1.67 (1.49, 1.87) | 1.71 (1.49, 1.96) | 1.73 (1.50, 2.00) |
| Physical frailty -proteomic signature | 1.60 (1.41, 1.82) | 1.53 (1.32, 1.77) | 1.52 (1.31, 1.77) |
| Psychological frailty-proteomic signature | 0.95 (0.83, 1.09) | 0.98 (0.85, 1.12) | 0.96 (0.83, 1.11) |
| **Sensitivity analysis 2 for asthma** |  |  |  |
| Frailty index | 2.09 (1.76, 2.47) | 1.95 (1.63, 2.33) | 1.85 (1.54, 2.23) |
| Physical frailty | 1.27 (1.07, 1.51) | 1.13 (0.95, 1.36) | 1.09 (0.91, 1.31) |
| Psychological frailty | 1.35 (1.14, 1.60) | 1.32 (1.11, 1.56) | 1.28 (1.08, 1.53) |
| Frailty index-proteomic signature | 1.27 (1.16, 1.38) | 1.22 (1.11, 1.35) | 1.12 (1.02, 1.24) |
| Physical frailty -proteomic signature | 1.22 (1.11, 1.33) | 1.11 (1.01, 1.23) | 1.10 (0.99, 1.22) |
| Psychological frailty-proteomic signature | 1.05 (0.97, 1.15) | 1.11 (1.01, 1.21) | 1.09 (0.99, 1.19) |
| **Sensitivity analysis 2 for COPD** |  |  |  |
| Frailty index | 2.37 (2.02, 2.80) | 2.03 (1.71, 2.42) | 1.64 (1.37, 1.96) |
| Physical frailty | 1.41 (1.20, 1.66) | 1.31 (1.10, 1.56) | 1.06 (0.89, 1.27) |
| Psychological frailty | 1.34 (1.14, 1.57) | 1.20 (1.02, 1.40) | 1.19 (1.01, 1.40) |
| Frailty index-proteomic signature | 1.70 (1.59, 1.82) | 1.65 (1.52, 1.79) | 1.55 (1.43, 1.69) |
| Physical frailty -proteomic signature | 1.67 (1.55, 1.79) | 1.62 (1.49, 1.76) | 1.61 (1.47, 1.75) |
| Psychological frailty-proteomic signature | 1.01 (0.93, 1.10) | 1.03 (0.95, 1.11) | 1.01 (0.93, 1.10) |
| **Sensitivity analysis 2 for IPF** |  |  |  |
| Frailty index | 1.21 (0.90, 1.61) | 1.06 (0.78, 1.44) | 0.83 (0.60, 1.15) |
| Physical frailty | 1.38 (1.03, 1.86) | 1.32 (0.97, 1.81) | 1.15 (0.84, 1.59) |
| Psychological frailty | 1.17 (0.87, 1.56) | 1.14 (0.85, 1.53) | 1.18 (0.88, 1.58) |
| Frailty index-proteomic signature | 1.59 (1.40, 1.81) | 1.60 (1.38, 1.87) | 1.64 (1.40, 1.92) |
| Physical frailty -proteomic signature | 1.53 (1.33, 1.75) | 1.45 (1.23, 1.70) | 1.42 (1.21, 1.68) |
| Psychological frailty-proteomic signature | 0.90 (0.78, 1.05) | 0.93 (0.80, 1.08) | 0.92 (0.79, 1.07) |
| **Sensitivity analysis 3 for asthma** |  |  |  |
| Frailty index | 1.96 (1.64, 2.34) | 1.88 (1.56, 2.27) | 1.80 (1.49, 2.19) |
| Physical frailty | 1.29 (1.08, 1.54) | 1.17 (0.97, 1.42) | 1.13 (0.93, 1.37) |
| Psychological frailty | 1.27 (1.06, 1.52) | 1.25 (1.05, 1.50) | 1.24 (1.03, 1.48) |
| Frailty index-proteomic signature | 1.23 (1.12, 1.34) | 1.20 (1.08, 1.33) | 1.11 (1.00, 1.23) |
| Physical frailty -proteomic signature | 1.21 (1.11, 1.33) | 1.13 (1.02, 1.26) | 1.11 (1.00, 1.24) |
| Psychological frailty-proteomic signature | 1.02 (0.93, 1.11) | 1.07 (0.97, 1.17) | 1.05 (0.96, 1.15) |
| **Sensitivity analysis 3 for COPD** |  |  |  |
| Frailty index | 2.40 (2.02, 2.86) | 2.08 (1.73, 2.49) | 1.68 (1.39, 2.03) |
| Physical frailty | 1.46 (1.23, 1.74) | 1.32 (1.10, 1.58) | 1.07 (0.88, 1.29) |
| Psychological frailty | 1.36 (1.15, 1.60) | 1.21 (1.02, 1.43) | 1.19 (1.01, 1.42) |
| Frailty index-proteomic signature | 1.69 (1.57, 1.81) | 1.65 (1.52, 1.80) | 1.56 (1.43, 1.70) |
| Physical frailty -proteomic signature | 1.71 (1.58, 1.84) | 1.65 (1.51, 1.80) | 1.64 (1.50, 1.80) |
| Psychological frailty-proteomic signature | 1.04 (0.95, 1.13) | 1.06 (0.97, 1.15) | 1.04 (0.96, 1.14) |
| **Sensitivity analysis 3 for IPF** |  |  |  |
| Frailty index | 1.21 (0.89, 1.63) | 1.02 (0.74, 1.41) | 0.77 (0.55, 1.08) |
| Physical frailty | 1.26 (0.93, 1.71) | 1.11 (0.81, 1.53) | 0.95 (0.68, 1.32) |
| Psychological frailty | 1.06 (0.78, 1.43) | 1.00 (0.74, 1.35) | 1.01 (0.74, 1.37) |
| Frailty index-proteomic signature | 1.69 (1.49, 1.92) | 1.71 (1.46, 1.99) | 1.76 (1.50, 2.07) |
| Physical frailty -proteomic signature | 1.59 (1.38, 1.82) | 1.47 (1.25, 1.72) | 1.48 (1.25, 1.74) |
| Psychological frailty-proteomic signature | 0.95 (0.81, 1.11) | 0.96 (0.82, 1.12) | 0.96 (0.82, 1.12) |
| **Sensitivity analysis 4 for asthma** |  |  |  |
| Frailty index | 1.97 (1.65, 2.34) | 1.87 (1.56, 2.25) | 1.78 (1.47, 2.15) |
| Physical frailty | 1.22 (1.03, 1.46) | 1.09 (0.91, 1.31) | 1.04 (0.86, 1.26) |
| Psychological frailty | 1.26 (1.06, 1.50) | 1.23 (1.03, 1.47) | 1.20 (1.01, 1.44) |
| Frailty index-proteomic signature | 1.25 (1.14, 1.36) | 1.22 (1.11, 1.36) | 1.13 (1.02, 1.26) |
| Physical frailty -proteomic signature | 1.23 (1.13, 1.35) | 1.14 (1.03, 1.27) | 1.14 (1.02, 1.26) |
| Psychological frailty-proteomic signature | 1.05 (0.96, 1.14) | 1.10 (1.00, 1.20) | 1.08 (0.99, 1.18) |
| **Sensitivity analysis 4 for COPD** |  |  |  |
| Frailty index | 2.48 (2.09, 2.94) | 2.04 (1.71, 2.45) | 1.66 (1.38, 2.00) |
| Physical frailty | 1.52 (1.28, 1.80) | 1.32 (1.10, 1.58) | 1.07 (0.89, 1.29) |
| Psychological frailty | 1.25 (1.06, 1.48) | 1.09 (0.92, 1.29) | 1.10 (0.93, 1.30) |
| Frailty index-proteomic signature | 1.74 (1.62, 1.86) | 1.66 (1.52, 1.81) | 1.56 (1.43, 1.71) |
| Physical frailty -proteomic signature | 1.75 (1.63, 1.88) | 1.62 (1.48, 1.77) | 1.61 (1.47, 1.76) |
| Psychological frailty-proteomic signature | 0.98 (0.90, 1.06) | 0.99 (0.91, 1.08) | 0.99 (0.91, 1.07) |
| **Sensitivity analysis 4 for IPF** |  |  |  |
| Frailty index | 1.55 (1.17, 2.04) | 1.35 (1.01, 1.82) | 1.05 (0.77, 1.43) |
| Physical frailty | 1.39 (1.05, 1.85) | 1.22 (0.90, 1.65) | 1.03 (0.76, 1.41) |
| Psychological frailty | 1.33 (1.01, 1.76) | 1.26 (0.95, 1.67) | 1.28 (0.97, 1.71) |
| Frailty index-proteomic signature | 1.68 (1.49, 1.90) | 1.71 (1.48, 1.98) | 1.70 (1.46, 1.98) |
| Physical frailty -proteomic signature | 1.62 (1.42, 1.84) | 1.52 (1.31, 1.77) | 1.51 (1.29, 1.77) |
| Psychological frailty-proteomic signature | 0.93 (0.81, 1.08) | 0.96 (0.83, 1.11) | 0.94 (0.81, 1.09) |
| **Sensitivity analysis 5 for asthma** |  |  |  |
| Frailty index | 1.92 (1.61, 2.29) | 1.84 (1.53, 2.22) | 1.77 (1.45, 2.15) |
| Physical frailty | 1.20 (1.01, 1.43) | 1.08 (0.90, 1.31) | 1.04 (0.86, 1.26) |
| Psychological frailty | 1.26 (1.06, 1.50) | 1.24 (1.04, 1.47) | 1.21 (1.01, 1.45) |
| Frailty index-proteomic signature | 1.21 (1.11, 1.32) | 1.19 (1.07, 1.32) | 1.10 (0.99, 1.23) |
| Physical frailty -proteomic signature | 1.19 (1.09, 1.30) | 1.11 (1.00, 1.23) | 1.10 (0.99, 1.22) |
| Psychological frailty-proteomic signature | 1.04 (0.95, 1.14) | 1.09 (1.00, 1.19) | 1.07 (0.98, 1.18) |
| **Sensitivity analysis 5 for COPD** |  |  |  |
| Frailty index | 2.41 (2.03, 2.86) | 1.98 (1.65, 2.38) | 1.65 (1.37, 2.00) |
| Physical frailty | 1.49 (1.25, 1.76) | 1.31 (1.09, 1.57) | 1.09 (0.91, 1.31) |
| Psychological frailty | 1.26 (1.07, 1.48) | 1.10 (0.93, 1.30) | 1.11 (0.94, 1.32) |
| Frailty index-proteomic signature | 1.65 (1.55, 1.76) | 1.57 (1.44, 1.70) | 1.48 (1.35, 1.61) |
| Physical frailty -proteomic signature | 1.64 (1.54, 1.76) | 1.52 (1.39, 1.65) | 1.50 (1.38, 1.64) |
| Psychological frailty-proteomic signature | 0.97 (0.89, 1.06) | 0.98 (0.89, 1.07) | 0.97 (0.89, 1.06) |
| **Sensitivity analysis 5 for IPF** |  |  |  |
| Frailty index | 1.49 (1.13, 1.97) | 1.31 (0.98, 1.76) | 1.04 (0.77, 1.41) |
| Physical frailty | 1.36 (1.02, 1.80) | 1.21 (0.89, 1.63) | 1.05 (0.77, 1.42) |
| Psychological frailty | 1.33 (1.01, 1.75) | 1.27 (0.96, 1.67) | 1.29 (0.98, 1.71) |
| Frailty index-proteomic signature | 1.60 (1.44, 1.77) | 1.62 (1.42, 1.84) | 1.61 (1.41, 1.84) |
| Physical frailty -proteomic signature | 1.52 (1.35, 1.72) | 1.44 (1.24, 1.66) | 1.43 (1.23, 1.66) |
| Psychological frailty-proteomic signature | 0.93 (0.79, 1.09) | 0.95 (0.81, 1.12) | 0.93 (0.79, 1.1) |

Abbreviations: MV, multiple variables; MA, mutual adjustment. **a** Sensitivity analysis 1 we restricted the analysis to Whites due to the high proportion (~97%) of Whites in the cohort; sensitivity analysis 2: to minimize reverse causation, individuals who developed CRDs within the first two years of follow-up were excluded; sensitivity analysis 3: to mitigate potential confounding effects of the COVID-19 pandemic, follow-up was censored on December 31, 2019; sensitivity analysis 4: we tested the influence of the definitions of confounding factors by replacing moderate drinking with never drinking, and replacing education, income, and IMD with socioeconomic status; sensitivity analysis 5: we employed the Fine-Gray model to account for competing risks. **b** Three models were applied to estimate the associations. The basic model adjusted for socio-demographic factors. The MV additionally adjusted for BMI, WHR, occupational exposure, and lifestyle factors, C-reactive proteins, environmental factors, and prevalent diseases. The mutually adjusted model (MV+MA) included both frailty phenotypes and its corresponding proteomic signature.
